# Immune gene expression profiling reveals heterogeneity in luminal breast tumors

**DOI:** 10.1186/s13058-019-1218-9

**Published:** 2019-12-19

**Authors:** Bin Zhu, Lap Ah Tse, Difei Wang, Hela Koka, Tongwu Zhang, Mustapha Abubakar, Priscilla Lee, Feng Wang, Cherry Wu, Koon Ho Tsang, Wing-cheong Chan, Sze Hong Law, Mengjie Li, Wentao Li, Suyang Wu, Zhiguang Liu, Bixia Huang, Han Zhang, Eric Tang, Zhengyan Kan, Soohyeon Lee, Yeon Hee Park, Seok Jin Nam, Mingyi Wang, Xuezheng Sun, Kristine Jones, Bin Zhu, Amy Hutchinson, Belynda Hicks, Ludmila Prokunina-Olsson, Jianxin Shi, Montserrat Garcia-Closas, Stephen Chanock, Xiaohong R. Yang

**Affiliations:** 1 Division of Cancer Epidemiology and Genetics, National Cancer Institute, National Institutes of Health, Rockville, MD USA; 2 Division of Occupational and Environmental Health, The Chinese University of Hong Kong, Hong Kong, China; 3 Cancer Genomics Research Laboratory, Leidos Biomedical Research, Frederick National Laboratory for Cancer Research, Frederick, MD USA; 4 North District Hospital, Hong Kong, China; 5 Yan Chai Hospital, Hong Kong, China; 6 Vanderbilt University, Nashville, TN USA; 7 Pfizer Oncology Research, San Diego, CA 92121 USA; 8 Pfizer Oncology, Seoul, 04631 South Korea; 9 Division of Hematology-Oncology, Department of Medicine, Samsung Medical Center, Sungkyunkwan University School of Medicine, Seoul, 06351 South Korea; 10 Department of Epidemiology, University of North Carolina at Chapel Hill, Chapel Hill, NC USA

**Keywords:** Tumor-infiltrating lymphocytes, Immune subtypes, Luminal breast cancer, Asian, Somatic mutations, *APOBEC3B* germline deletion

## Abstract

**Background:**

Heterogeneity of immune gene expression patterns of luminal breast cancer (BC), which is clinically heterogeneous and overall considered as low immunogenic, has not been well studied especially in non-European populations. Here, we aimed at characterizing the immune gene expression profile of luminal BC in an Asian population and associating it with patient characteristics and tumor genomic features.

**Methods:**

We performed immune gene expression profiling of tumor and adjacent normal tissue in 92 luminal BC patients from Hong Kong using RNA-sequencing data and used unsupervised consensus clustering to stratify tumors. We then used luminal patients from The Cancer Genome Atlas (TCGA, *N* = 564) and a Korean breast cancer study (KBC, *N* = 112) as replication datasets.

**Results:**

Based on the expression of 130 immune-related genes, luminal tumors were stratified into three distinct immune subtypes. Tumors in one subtype showed higher level of tumor-infiltrating lymphocytes (TILs), characterized by T cell gene activation, higher expression of immune checkpoint genes, higher nonsynonymous mutation burden, and higher *APOBEC*-signature mutations, compared with other luminal tumors. The high-TIL subtype was also associated with lower *ESR1/ESR2* expression ratio and increasing body mass index. The comparison of the immune profile in tumor and matched normal tissue suggested a tumor-derived activation of specific immune responses, which was only seen in high-TIL patients. Tumors in a second subtype were characterized by increased expression of interferon-stimulated genes and enrichment for *TP53* somatic mutations. The presence of three immune subtypes within luminal BC was replicated in TCGA and KBC, although the pattern was more similar in Asian populations. The germline *APOBEC3B* deletion polymorphism, which is prevalent in East Asian populations and was previously linked to immune activation, was not associated with immune subtypes in our study. This result does not support the hypothesis that the germline *APOBEC3B* deletion polymorphism is the driving force for immune activation in breast tumors in Asian populations.

**Conclusion:**

Our findings suggest that immune gene expression and associated genomic features could be useful to further stratify luminal BC beyond the current luminal A/B classification and a subset of luminal BC patients may benefit from checkpoint immunotherapy, at least in Asian populations.

## Background

Breast cancer (BC) is a heterogeneous disease comprised of several molecular subtypes (luminal A, luminal B, HER2-enriched, and basal-like) with distinct molecular features and clinical behaviors [1, 2]. Within each subtype, substantial heterogeneity still exists in terms of genomic features and clinical outcomes, particularly in luminal BC [3–5]. The commonly used luminal A/B classification based on proliferation does not fully capture heterogeneity in luminal tumors [6, 7]. A recent study [8] partitioned luminal breast tumors of The Cancer Genome Atlas (TCGA) into two distinct prognostic subgroups that exhibited differential expression of immune-related genes. This partition showed better discriminative prognostic value than the luminal A/B classification, suggesting that the immunogenicity of luminal tumors is heterogeneous.

The investigation of tumor-infiltrating lymphocytes (TILs) has greatly improved our knowledge of the nature of tumor-immune interactions. The presence of TILs has been associated with a favorable prognosis across multiple cancer types including BC. Recently, data from a clinical trial of triple-negative breast cancer (TNBC) patients demonstrated that the combination of immunotherapy with chemotherapy was associated with improved patient outcomes [9], which led to the first approval of checkpoint immunotherapy in BC by the Food and Drug Administration. However, TILs might be associated with treatment responses and survival in a subtype-specific manner [10, 11]. Recent TCGA Pan-Cancer studies identified substantial heterogeneity in immune profiles across and within cancer types as well as within cancer subtypes [12, 13]. For example, Thorsson et al. [12] identified six immune subtypes spanning multiple cancer types and most breast tumors fell into three of these immune subtypes. Among BC molecular subtypes, luminal-A tumors showed the greatest heterogeneity, with a similar number of tumors classified into each of the three immune subtypes. Nevertheless, variation in immune profiles within luminal tumors may not be sufficiently characterized in these Pan-Cancer analyses since the immune stratification was likely driven by high-TIL tumor types/subtypes [14]. A more detailed understanding of the variation in TILs among luminal tumors could provide new insights into luminal BC heterogeneity and identify a subset who might be amenable to immunomodulation and benefit from immunotherapy.

So far, most studies that conduct profiles of immune cells in BC have used data from TCGA, which does not represent the general patient population, particularly for non-European subjects. Previous studies have shown that tumor immunobiology might vary by race/ethnicity [15, 16] and different germline genetic architecture may play a role but how germline variants contribute to immune phenotype has not been extensively studied. For example, the germline *APOBEC3B* deletion polymorphism, which is more common in East Asians (31.2%) than in Europeans (9.0%) and West Africans (4.2%) based on HapMap, is not well represented in TCGA. This deletion has been associated with increased BC risk [17] and immune gene expression [18, 19], suggesting that East Asian BCs may exhibit a distinct immune profile compared to other BC populations. In this study, we profiled immune gene expression in paired tumor/normal luminal breast tissue collected from a hospital-based case-control study of Asian BC patients in Hong Kong (HKBC), for whom extensive clinical and epidemiologic data were collected.

## Methods

### Participants and samples

We analyzed data and biospecimens collected from a hospital-based BC case-control study in Hong Kong as previously described [20]. In brief, fresh frozen breast tumors and paired normal tissues were collected from newly diagnosed BC patients in two HK hospitals between 2013 and 2016. Patients with pre-surgery treatment were excluded from the study. Clinical characteristics and BC risk factors were obtained from medical records and questionnaires. The study protocol was approved by ethics committees of the Joint Chinese University of Hong Kong-New Territories East Cluster, the Kowloon West Cluster, and the National Cancer Institute (NCI). Written informed consent was obtained prior to the surgery for all participants.

### Genomic and bioinformatic analyses

Paired tumor and histologically normal breast tissue samples were processed for pathology review at the Biospecimen Core Resource (BCR), Nationwide Children’s Hospital, using modified TCGA criteria [21]. Specifically, only tumors with > 50% tumor cells and normal tissue with 0% tumor cells were included for DNA/RNA extraction.

RNA sequencing (RNA-Seq) data were generated in 139 tumors and 92 histologically normal breast tissue samples that passed on all quality control metrics at Macrogen Corporation on Illumina HiSeq4000 using TruSeq stranded RNA kit with Ribo-Zero for rRNA depletion and 100-bp paired-end method. Gene expression was quantified as TPM (transcript per million) using RSEM [22], and log_2_TPM was used for statistical analyses. PAM50 subtype was defined by an absolute intrinsic subtyping (AIMS) method [23]. Three computational algorithms were used to characterize immune cell composition in both tumor and paired normal breast tissue: ESTIMATE [24], CIBERSORT [25], and MCP-counter [26]. While ESTIMATE (for overall infiltration of immune cells) and MCP-counter (for eight immune cell subpopulations) both measure the abundance of immune cells in a given sample, CIBERSORT estimates intra-sample proportions of 23 immune cell subpopulations.

Whole-exome sequencing (WES) was performed on 104 paired tumor and normal samples (59 of them also had RNA-Seq data) at the Cancer Genomics Research Laboratory (CGR), NCI, using SeqCAP EZ Human Exome Library v3.0 (Roche NimbleGen, Madison, WI) for exome sequence capture. The captured DNA was then subjected to paired-end sequencing utilizing Illumina HiSeq2000. The average sequencing depth was 106.2x for tumors and 47.6x for the paired normal tissues. Somatic mutations were called using four callers, and the analyses were based on mutations called by three or more of four established callers (MuTect [27], MuTect2 (GATK tool), Strelka [28], and TNScope by Sentieon [29]).

SNP rs12628403, which is a proxy for the *APOBEC3B* deletion (*r*^2^ = 1.00 in Chinese from Beijing (CHB) in HapMap samples), was genotyped in germline DNA with a custom TaqMan assay as previously described [30].

### TIL assessment based on pathology review

We assembled hemotoxylin and eosin (H&E)-stained frozen sections from the same frozen tumors used for DNA/RNA extraction and formalin-fixed paraffin-embedded (FFPE) sections from the same set of HK patients. Using the Halo image analysis platform (Indica Labs, Albuquerque, New Mexico), we developed a multi-step approach for the quantification of TILs which was based on supervised machine learning analysis of histological images (Additional file 1: Figure S1). In the first step, we trained an algorithm to segment the tumor into epithelial, stromal, and adipose tissue regions (panel B, Additional file 1: Figure S1). Next, we trained a cell-detection algorithm to identify TILs based on contexture (nuclear detection weight = 0.35; nuclear contrast threshold = 0.54), size (5–20 μm) and shape (minimum nuclear roundness = 0.45) (panel E, Additional file 1: Figure S1) within well-defined regions of interest. By focusing on the stroma (intra-tumoral and peri-tumoral; panels E and F Additional file 1: Figure S1), we then applied this algorithm to the centralized evaluation of TILs in all images.

### Replication datasets

We analyzed two available, independent datasets to replicate our findings: 564 luminal patients in TCGA [3] and 112 luminal patients in a Korean BC genomic study (KBC) [31]. We analyzed TCGA Asians (*n* = 29, mean age: 51 years), African Americans (AA, *n* = 72, mean age 58 years), and European ancestry (EA, *n* = 463, mean age 60 years) separately. PAM50 was called using the same AIMS method for each TCGA sample as it was used in HKBC. KBC patients were much younger, with a mean age at diagnosis of 40 years. PAM50 subtype and mutation calling for KBC were previously detailed [31]. Immune classification and composition across all datasets (HKBC, TCGA, and KBC) were analyzed using the same methods.

### Statistical analysis

The consensus clustering was conducted using ConsensusClusterPlus [32], based on expression of 130 immune-related genes (within 13 previously reported metagenes, including T cell signatures, activated CD8/NK cells, interferon-stimulated genes, and etc., Additional file 2: Table S2) [33]. Expression levels of these metagenes correspond to activities of various types of immune cells and reflect various immune functions. The prognostic and predictive values of these metagenes have been previously assessed in TCGA and other independent datasets [34, 35]. For each of the 500 resampling of subjects, we sampled 80% of the subjects and clustered them using agglomerative hierarchical clustering with Pearson correlation as the distance metric. We evaluated up to 10 clusters and chose 3 clusters (*k* = 3) as they fit the data best.

A comprehensive characterization of immune cell composition in both tumor and paired normal breast tissue was achieved by using three computational algorithms: ESTIMATE [24], CIBERSORT [25], and MCP-counter [26]. The ANOVA test was used to compare mean differences across the luminal immune subtypes for immune cell populations and their immune scores. Logistic regression was used to assess the associations between the immune subtypes (outcome) and genomic alterations, patient characteristics, and BC risk factors, with the adjustment for age at diagnosis and body mass index (BMI). The Kaplan–Meier method was used to assess overall survival among patients, stratified by immune subtypes. A multivariable Cox proportional hazards model was also used to test the differences in survival across immune subtypes with the adjustment of age at diagnosis and tumor stage. Since most of our analyses were exploratory, we did not adjust for multiple testing. All statistical tests were two-sided and performed using SAS version 9.3 (SAS Institute, Cary, NC, USA) or R version 3.4.4 (R Foundation for Statistical Computing, Vienna, Austria).

## Results

The analysis included 92 luminal tumors and 56 normal samples from HKBC (including 56 tumor/normal tissue pairs). The mean age at diagnosis was 58.7 years, and 49 (53.3%) patients were classified as luminal A according to PAM50. Although our analyses were focused on luminal patients, we also present data for HER2-enriched and basal-like patients as a comparison group (*n* = 40). The distribution of clinical characteristics and key BC risk factors among these patients is shown in Additional file 2: Table S1.

### Immune gene expression stratified luminal tumors into three subtypes

**Fig. 1:**
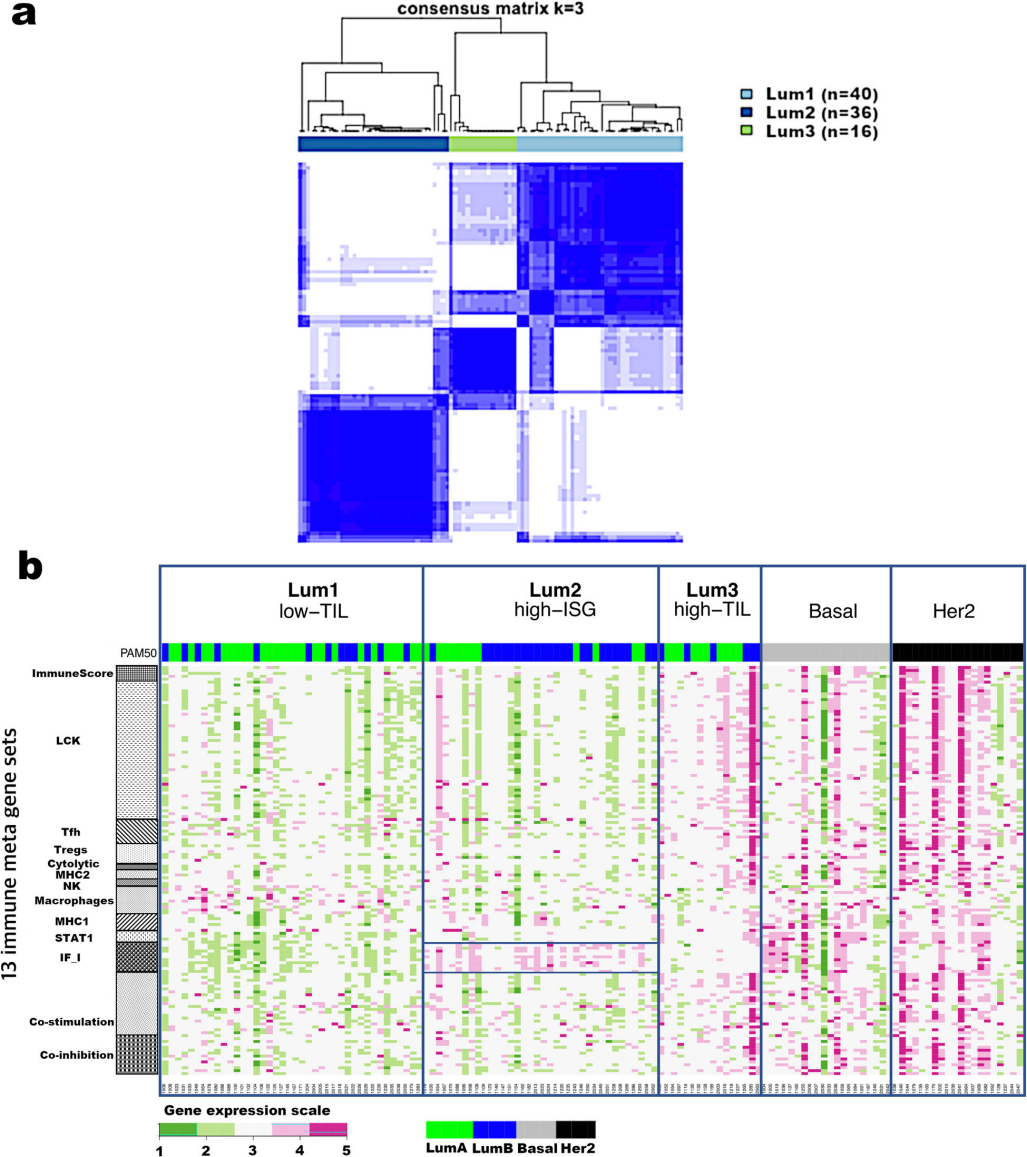
Consensus clustering of 92 luminal breast tumors from Hong Kong patients based on 130 immune-related genes. **a** Consensus cluster matrix showing three major clusters. **b** Gene expression heatmap showing gene expression levels of 13 immune metagenes in the three luminal immune subtypes (low-TIL, high-ISG, and high-TIL) and in non-luminal (HER2-enriched and basal-like) tumors. Each column represents a patient, grouped by immune subtypes; each row represents a gene, grouped by 13 immune pathways. Normalized gene expression value with mean = 0 and standard deviation (SD) = 1 is indicated by 5 color categories representing the increasing expression level from green to red. LCK lymphocyte-specific protein tyrosine kinase, Tfh helper follicular T cell, Tregs regulatory T cell, NK natural killer cell, MHC major histocompatibility complex, STAT1 signal transducer and activator of transcription 1, IF_I interferon inducible genes (boxed for Lum2/high-ISG); PAM50: green = luminal A, blue = luminal B, gray = basal, black = HER2-enriched

**Fig. 2:**
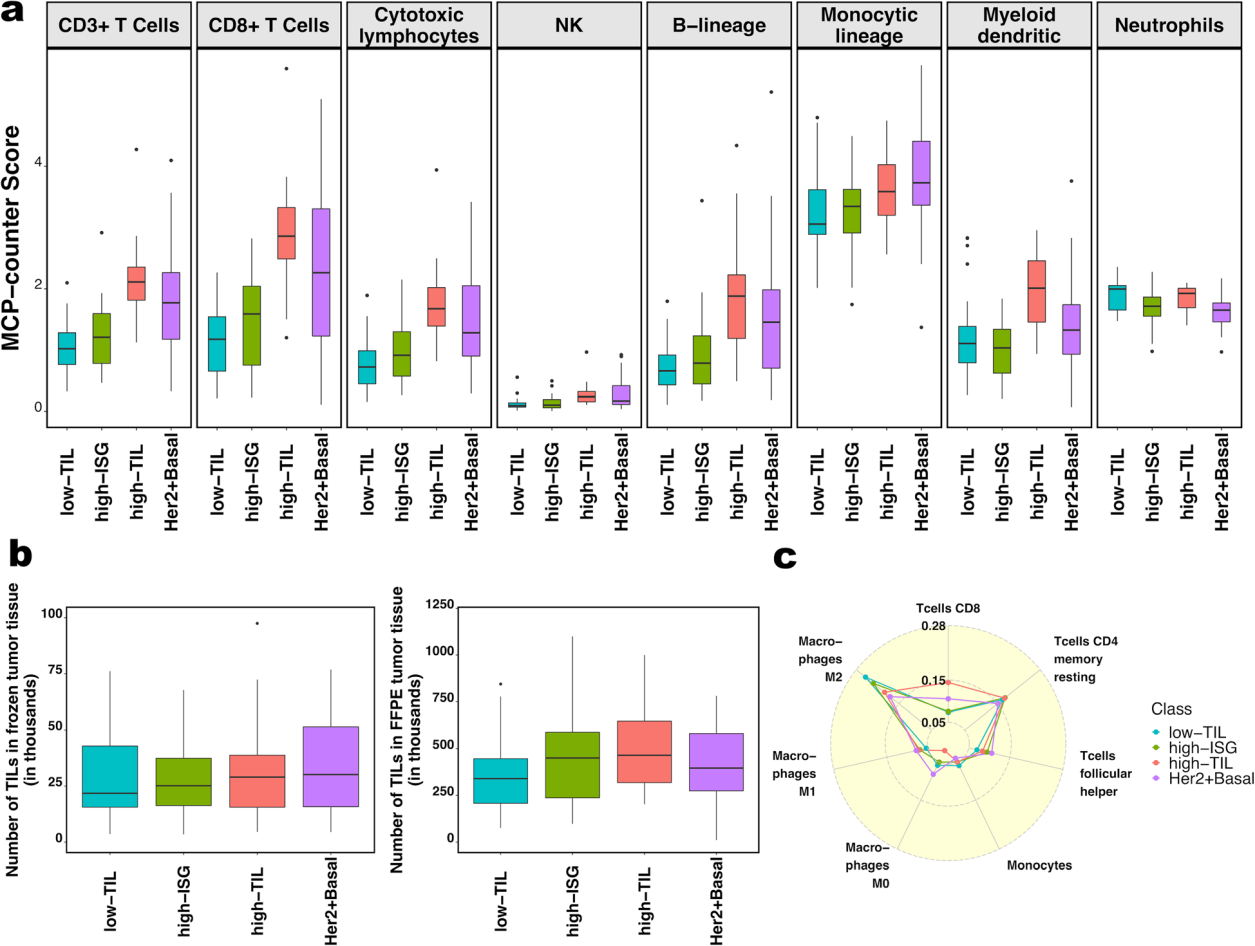
The immune phenotype in the three luminal immune subtypes (low-TIL, high-ISG, and high-TIL) and in non-luminal (HER2-enriched and basal-like) tumors. **a** Abundance of eight immune cell subpopulations (estimated by MCP-counter). **b** Number of TILs in frozen (left) and formalin-fixed paraffin-embedded (FFPE) tumors based on pathology assessment of H&E sections. **c** Relative fractions of immune cell populations (inferred by CIBERSORT). Immune cell populations with low fractions (average < 10% across all samples) are not shown

We conducted unsupervised consensus clustering of 92 luminal tumors using expression of 130 immune-related genes. The best separation was achieved by dividing the luminal patients into three subtypes (lum1: *n* = 40; lum2: *n* = 36; lum3; *n* = 16; Fig. 1a); lum1 and lum3 were enriched with luminal-A tumors and lum2 enriched with luminal-B tumors (Additional file 2: Table S3). Lum1 expressed low levels of most immune genes (Fig. 1b) and therefore was designated as low-TIL. Lum2 had high expression of *STAT1* and other interferon-stimulated genes (ISGs), but low expression of other immune genes (Fig. 1b), designated as high-ISG. Lum3 (defined as high-TIL) showed the highest expression level of most immune genes (Fig. 1b) such as immune checkpoint genes (e.g., *PD-L1* and *CTLA-4*), chemokine genes and their receptors (e.g., *CXCL9* and *CXCL10*), and effectors (e.g., *GZMK* and *PRF1*) (Additional file 1: Figure S2), reflecting a T cell-inflamed phenotype. Compared to low-TIL and high-ISG tumors, high-TIL tumors had higher abundance of most immune subpopulations (estimated by MCP-counter, Fig. 2a), except for neutrophils and cells of monocytic lineage. The abundance score for each immune subpopulation in high-TIL luminal tumors was comparable to that of HER2-enriched and basal-like tumors (Fig. 2a; Additional file 1: Figure S3; *P* values see Additional file 2: Table S4). Adjusting for tumor purity, which was inferred using ESTIMATE purity score, did not change the results (Additional file 1: Figure S4). The results from TIL assessment based on H&E staining of frozen and FFPE sections were consistent, confirming that TILs were more abundant in tumor stroma in high-TIL versus low-TIL patients (Fig. 2b).

We also inferred the fractions of 23 immune cell subpopulations in these patients using CIBERSORT, which estimates the relative fraction of each cell population in a sample rather than the absolute abundance. Figure 2c shows the fractions of seven subpopulations with the average fraction > 10% across all samples. We found that high-TIL tumors showed higher fractions of CD8+ T cells and tumor-killing M1 macrophages [36] than those of low-TIL and high-ISG tumors, while they had lower frequencies of tumor-promoting M2 and undifferentiated M0 macrophages (*P* values see Additional file 2: Table S5).

### The presence of luminal immune subtypes was replicated in independent studies

**Fig. 3:**
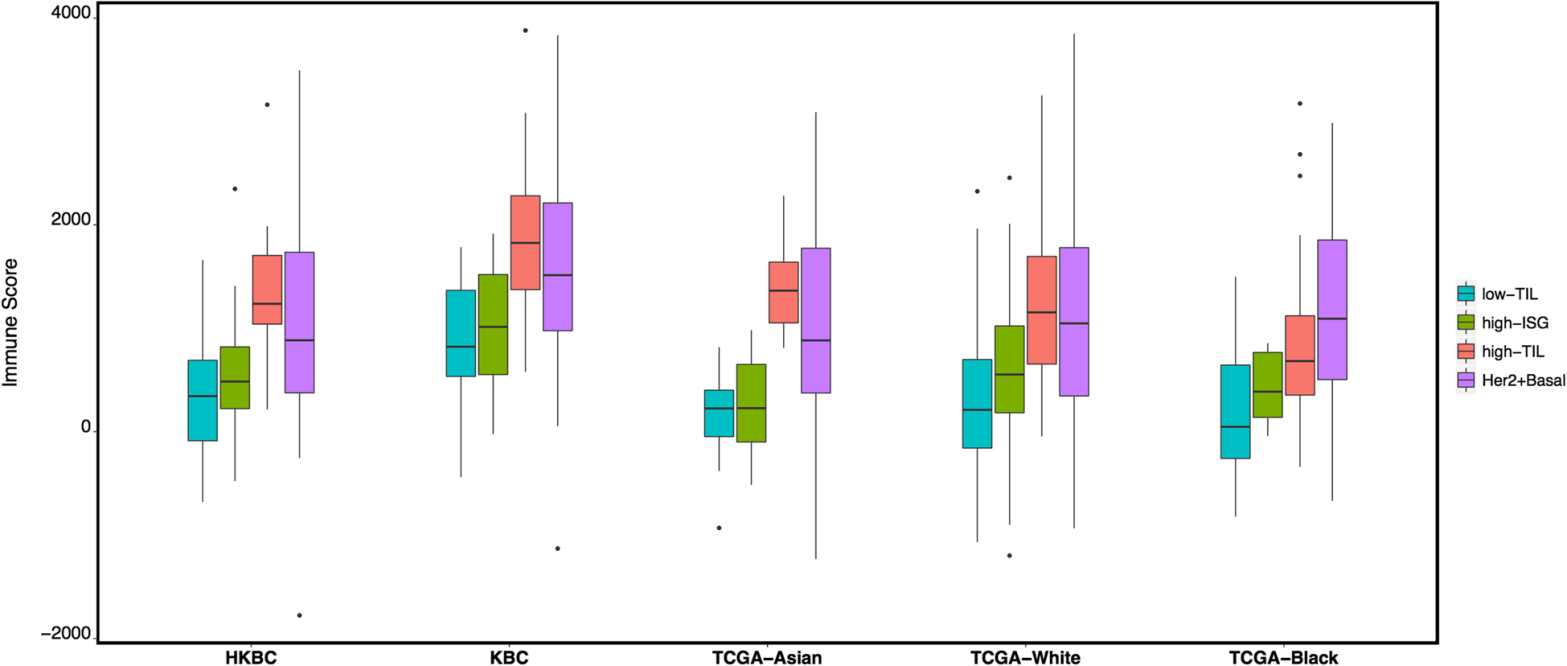
Average immune scores (inferred by ESTIMATE) in the three luminal immune subtypes and non-luminal (HER2-enriched and basal-like) tumors in HKBC, KBC, and TCGA (Asian, African American, and White, separately) datasets

Based on expression levels of the same 130 immune genes used in HKBC, luminal tumors in each TCGA population (Asian, African American, and White) and KBC were similarly assigned to three subtypes using consensus clustering, with the presence of a high-TIL luminal subtype seen in all populations (Fig. 3). The pattern was more similar in the three Asian populations, with a more pronounced separation of the high-TIL subtype from the other two subtypes. Consistent with HKBC results, high-TIL tumors in all replication datasets showed higher overall immune score (by ESTIMATE, Fig. 3), higher abundance of most immune subpopulations (by MCP-counter, Additional file 1: Figure S5a), and higher fractions of CD8+ T cells and M1 macrophages (by CIBERSORT, Additional file 1: Figure S5b). Like HKBC, high-TIL tumors showed upregulation of genes in immune activation and regulation activities (Additional file 1: Figure S5c), while high-ISG tumors expressed higher levels of ISGs (e.g., *DDX58*) than tumors in the other two luminal immune subtypes (Additional file 1: Figure S5d).

### Clinical characteristics, BC risk factors, and genomic features associated with immune subtypes

**Fig. 4:**
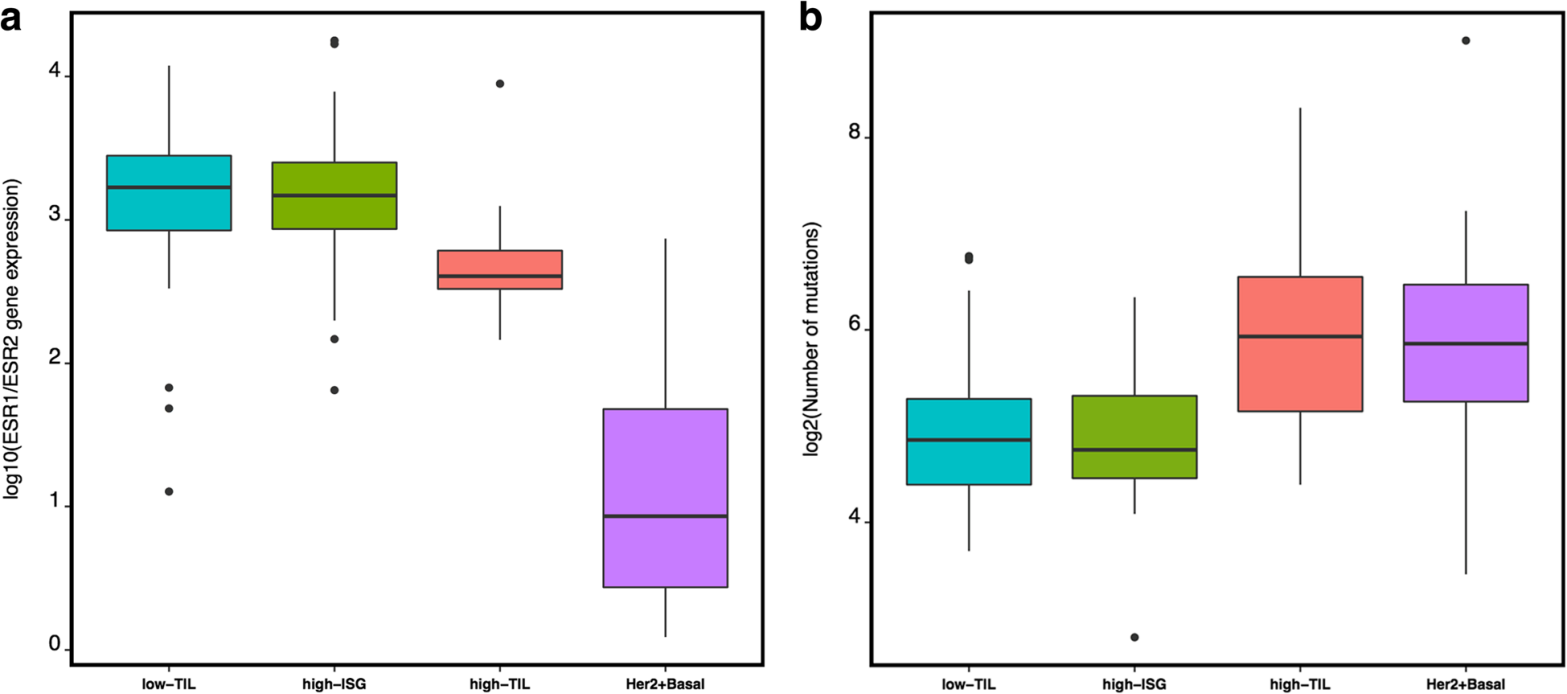
Genomic features associated with different immune subgroups. **a** *ESR1* and *ESR2* expression ratio (log scale). **b** Nonsynonymous mutation burden (log scale)

In HKBC, most clinical characteristics or BC risk factors examined, such as tumor grade, nodal status, age at menarche, parity, age at first birth, breastfeeding, and age at menopause, did not vary significantly across immune subtypes (Additional file 2: Table S6). However, the average BMI was higher in high-TIL (mean = 27.9) than in low-TIL (mean = 24.1) and high-ISG patients (mean = 24.6). The differences remained significant after the adjustment of age, menopausal status, and tumor purity (*P* = 0.0018 for high-TIL vs. low-TIL and *P* = 0.0057 for high-TIL vs. high-ISG). In addition, high-TIL tumors had slightly lower *ESR1* (estrogen receptor alpha) but higher *ESR2* (estrogen receptor beta) expression levels, resulting in a lower *ESR1*/*ESR2* ratio (*P* = 0.001) compared with low-TIL and high-ISG tumors (Fig. 4a, Additional file 1: Figure S6a). The association between the low *ESR1/ESR2* ratio and the high-TIL subtype was consistently seen in all TCGA populations (Additional file 1: Figure S7a).

High-TIL patients tended to be younger than patients with low-TIL tumors in HKBC as well as in the replication datasets (Additional file 1: Figure S7b), although the significant difference was only seen among the TCGA Whites (*P* = 0.018). The short follow-up time in HKBC prohibited us from assessing the prognostic outcome in relation to the immune subtypes. We therefore conducted survival analysis using TCGA data of 905 BC patients. We combined all ethnicity groups because few deaths occurred among Asian or African American patients. As shown in Additional file 1: Figure S7c, the high-TIL subtype was associated with the best 10-year overall survival among all subtypes (*P* = 0.008), although the difference became non-significant after the adjustment for age at diagnosis and stage (hazard ratio [HR] = 0.6, 95% confidence interval [CI] = 0.26–1.4, *P* = 0.22). The attenuation of the significance was likely due to younger ages in the high-TIL subtype as stage did not differ across luminal immune subtypes (*P* = 0.72).

To evaluate the possible contribution of germline variation in *APOBEC3B* to immune profiles and mutational events, we genotyped a SNP (rs12628403) that is a proxy for the *APOBEC3B* deletion in germline DNA [30]. In HKBC, the frequency of the rs12628403-C allele that tags the 30-kb deletion (44.7% among 76 luminal patients and 40.4% among all 114 patients with genotyping data) was similar to what was reported in East Asian populations [17]. We found the expected associations between the *APOBEC3B* deletion and decreased levels of *APOBEC3B* expression in both tumor and normal tissue, validating SNP rs12628403 as a proxy for *APOBEC3B* deletion (Additional file 1: Figure S8). The frequency of the deletion allele did not vary significantly by immune subtypes, either in HKBC or in TCGA White (Table 1). In addition, the expression level of *APOBEC3A_B*, which is a hybrid transcript resulting from the *APOBEC3B* deletion, did not vary significantly by luminal immune subtypes (*P* = 0.36). Further, the ESTIMATE immune scores did not vary across different genotypes of SNP rs12628403 (*P* = 0.56). Similar results were obtained in the analysis based on all tumor subtypes. In TCGA Whites, the homozygous deletion of *APOBEC3B* was very rare; only 2 of 329 luminal patients with genotyping data were homozygote and neither of them was in the high-TIL subtype (Table 1).

**Table 1:** The distribution of rs12628403 genotype by tumor subtypes in the Hong Kong breast cancer study (HKBC) and TCGA white population

		Low-TIL, *N* (%)	High-ISG, *N* (%)	High-TIL, *N* (%)	HER2+basal, *N* (%)
HKBC	A/A	10 (29.4%)	9 (32.2%)	3 (21.4%)	15 (46.9%)
	A/C	17 (50.0%)	15 (53.5%)	8 (57.2%)	13 (40.6%)
	C/C	7 (20.6%)	4 (14.3%)	3 (21.4%)	4 (12.5%)
	*P* value*	Ref	0.89	0.92	0.35
TCGA White	A/A	111 (90.3%)	117 (86.0%)	60 (85.7%)	158 (92.9%)
	A/C	11 (8.9%)	18 (13.3%)	10 (14.3%)	10 (5.9%)
	C/C	1 (0.8%)	1 (0.7%)	0 (0%)	2 (1.2%)
	*P* value*	Ref	0.66	0.44	0.62

*P values were obtained from Fisher’s exact test by comparing each immune subtype with low-TIL

In an exploratory analysis of a subset of luminal tumors with both RNA-Seq and WES data (*n* = 59), we found that, after age and BMI adjustment, high-TIL tumors were associated with a higher nonsynonymous mutation burden (*P* = 0.03 compared to low-TIL tumors, Fig. 4b, Additional file 1: Figure S6b) and a higher frequency of *APOBEC*-signature mutations (mean 23.6%) compared with low-TIL (7.6%, *P* = 0.045) and high-ISG (8.3%, *P* = 0.089) tumors. Notably, all *TP53* mutations (*n* = 8, Table 2) observed among luminal patients occurred in high-ISG tumors. The similar enrichment of *TP53* mutations in high-ISG tumors was also seen in TCGA Whites (*P* = 0.0064, Table 2). The frequency of *PIK3CA* mutations did not vary significantly by immune subtypes in HKBC but showed a slight increase in high-TIL tumors in TCGA Whites (*P* = 0.031 compared to low-TIL tumors).

**Table 2:** Frequency of nonsynonymous TP53 mutations by tumor subtypes in the Hong Kong breast cancer study (HKBC) and TCGA white population

		Low-TIL, *N* (%)	High-ISG, *N* (%)	High-TIL, *N* (%)	HER2+basal, *N* (%)
HKBC	WT	29 (100.0%)	11 (57.9%)	11 (100.0%)	10 (45.5%)
	MUT	0 (0%)	8 (42.1%)	0 (0%)	12 (54.5%)
	*P* value*	Ref	0.0002	–	< 0.0001
TCGA White	WT	169 (95.5%)	142 (87.1%)	83 (91.2%)	105 (50.7%)
	MUT	8 (4.5%)	21 (12.9%)	8 (8.8%)	102 (49.3%)
	*P* value*	Ref	0.01	0.18	< 0.0001

*P values were obtained from Fisher’s exact test by comparing each immune subtype with low-TIL

### Comparison to matched normal tissue suggested T cell activation in high-TIL tumors only

**Fig. 5:**
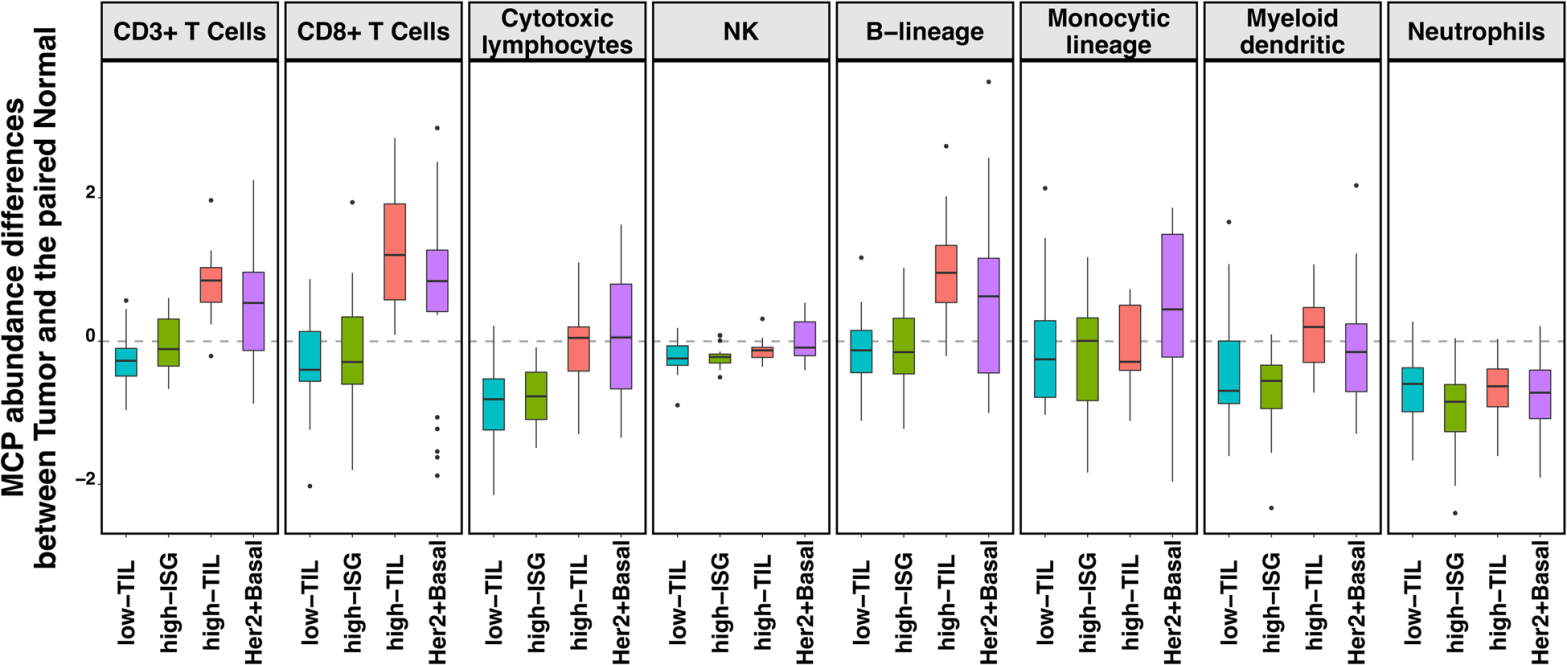
The mean differences in the abundance of eight immune cell subpopulations (estimated by MCP-counter) between paired tumor and normal tissue (T-N, *N* = 80) for the three luminal immune subtypes and non-luminal (HER2-enriched and basal-like) patients in HKBC, respectively. 0, no difference; > 0, higher in tumor than normal tissue; < 0, lower in tumor than in normal tissue

In our HKBC data, neither abundance nor fractions of the examined immune cell populations in paired normal breast tissue varied significantly across the three luminal immune subtypes (Additional file 1: Figure S9), suggesting that the distinguishing TIL levels between high-TIL and other tumors were not driven by the differences in their systematic normal TIL levels. Compared to matched normal (N) tissue, low-TIL and high-ISG tumors showed either no change or lower abundance of immune cell populations (such as cytotoxic lymphocytes), whereas high-TIL, like non-luminal tumors, had higher abundance scores of CD3+ T cells, CD8+ T cells, and B lineage cells (T-N difference > 0, Fig. 5; *P* value of CD8+ T cells = 0.0002 and 0.025 for high-TIL and non-luminal patients; other *P* values see Additional file 2: Table S7). These observations indicate a tumor-derived activation of specific immune responses in high-TIL and non-luminal tumors but not in other luminal tumors.

## Discussion

In this study, we identified three immune subtypes of luminal breast tumors in different BC genomic datasets. One luminal subtype (high-TIL) exhibited an activated immune phenotype and higher mutation burden that are similar to that of non-luminal (HER2-enriched and basal-like) tumors. Another luminal subtype (high-ISG) was characterized by increased expression of ISGs and enrichment for *TP53* mutations. These subtypes were consistently seen in independent datasets consisting of data based on different populations. Our findings suggest that immune gene expression and associated genomic features may reveal additional heterogeneity in luminal BC patients beyond the current luminal A/B classification, which may have implications for precision immunotherapy in luminal BC patients.

Previous studies suggested that the high expression of an alternative ER isoform, *ESR2* (encoding ERβ), was associated with favorable BC prognosis and that the association might depend on the ratio of *ESR1* and *ESR2* (ERα and ERβ) [37, 38]. Consistently, we observed that patients with increasing *ESR1/ESR2* ratio tended to have poorer survival (HR = 1.5, 95% CI = 0.7–3.3, *P* = 0.27, adjusting for age and stage) in TCGA luminal patients. Interestingly, in the current study, we found that high-TIL tumors had a lower *ESR1/ESR2* ratio as compared to low-TIL and high-ISG tumors in both HKBC and replication datasets. Our findings suggest that *ESR* expression, particularly *ESR2* expression, may relate to immune gene regulations in luminal breast tumors and this association may explain the previously reported favorable prognosis associated with ERβ expression.

We also identified a unique high-ISG luminal subtype that was enriched with luminal-B tumors and *TP53* mutations. Previous studies demonstrated that *TP53* mutations were associated with an immune-activated phenotype when all molecular subtypes were analyzed together, which is expected since *TP53* mutations are more prevalent in non-luminal than in luminal tumors. Our data suggest that *TP53* mutations may be specifically related to the activation of IFN-signaling, which was replicated in TCGA EA, suggesting that the relationship between immune composition and genomic determinants might be more complex than we previously appreciated.

In our study, we did not find a significant association between the germline *APOBEC3B* deletion and luminal immune subtypes. Similarly, the immune scores did not vary significantly by the deletion genotype, either in luminal or in all patients. The previously observed association between the deletion and immune activation was based on data from TCGA and METABRIC, in which the frequency of the homozygous deletion was very low [18, 19] and the results were driven by comparing the heterozygotes to the wild type. Although our evaluation was limited by the overall small sample size, the higher frequency of the deletion in this Asian population allowed us to examine both heterozygous and homozygous genotypes. Results based on our study do not support the hypothesis that the germline *APOBEC3B* deletion polymorphism is the driving force for immune activation in breast tumors [18, 19].

Taking advantage of our rich collection of epidemiologic data in HKBC, we examined several established BC risk factors in relation to the immune subtypes and found an association between higher BMI and the high-TIL luminal subtype. The average BMI was more than 3 units higher in high-TIL patients compared with other luminal patients, and the differences remained significant after the adjustment for potential confounders. Consistent with our finding in HKBC, a recent study reported a significant association between higher expression of CD8+ T cell signatures and increasing BMI in 1154 BC patients from the Nurses’ Health Study [39]. The link between obesity and BC involves multiple mechanisms that may interplay with each other such as chronic inflammation, estrogen production, growth factor stimulation, and altered metabolism [40]. Future large studies are warranted to follow up this observation.

In contrast to tumors, immune gene expression in adjacent normal tissue did not vary significantly across the three luminal immune subtypes, suggesting that high-TIL patients did not have high background immune activation. We found that high-TIL patients showed higher levels of CD3+ and CD8+ T cell in their tumors compared with normal tissues, which is similar to what was previously reported for ER-negative tumors [41]. These findings suggest that tumor-intrinsic events might drive the immune activation in a similar manner in ER-negative and high-TIL luminal tumors. In fact, consistent with what was reported by several previous studies [42, 43], we found that higher burden of nonsynonymous mutations and *APOBEC*-signature mutations might act as potential contributors to the increased immune response.

The strengths of our study include a comprehensive collection of clinical and exposure information and a detailed evaluation of immune composition for both tumors and paired normal tissue in an Asian population, and the replication of findings in independent datasets. The major limitation is the small sample size, which limited the power to identify genomic determinants of distinct immune phenotypes. Additionally, since we collected frozen breast tissue from recently diagnosed patients, the follow-up time is insufficient to evaluate the associations between the immune subtypes with prognostic outcomes. Large TIL studies of luminal BC with treatment and outcome data are warranted to follow up on our findings.

## Conclusions

In summary, we identified three immune subtypes of luminal breast tumors displaying distinct patterns of immune gene expression with associated genomic features in an Asian population. If confirmed, these findings may have important clinical implications in improving luminal BC stratification for precision oncology treatment [1, 5, 10, 11, 44].

## Supplementary information

**Additional file 1: Figure S1.** Schematic representation of the strategy for the quantification of TILs in H&E stained images. **Figure S2.** Expression levels of immune-related genes in the three luminal immune subtypes and non-luminal (HER2-enriched and basal-like) tumors in HKBC. **Figure S3.** MCP-counter scores and CIBERSORT relative fractions of a subset of immune cell subpopulations in the three luminal immune subtypes and non-luminal (HER2-enriched and basal-like, separately) tumors in HKBC. **Figure S4.** MCP-counter scores of eight immune cell subpopulations in the three luminal immune subtypes and non-luminal (HER2-enriched and basal-like) tumors with the adjustment of tumor purity in HKBC. **Figure S5.** Replication of luminal immune subtypes in TCGA and KBC datasets: a) MCP-counter scores for eight immune cell subpopulations; b) Relative fractions of immune cell subpopulations by CIBERSORT (cell populations with extremely low fractions were not shown); c) High-TIL tumors showed upregulation of genes in immune activation and regulation activities than tumors in the other two luminal immune subtypes; d) High-ISG tumors expressed higher levels of ISG genes than tumors in the other two luminal immune subtypes. **Figure S6.** Genomic features associated with luminal immune subtypes in HKBC (HER2-enriched and basal-like, separately): a) *ESR1*/*ESR2* ratios; b) number of mutations. **Figure S7.** Replication of genomic features associated with luminal immune subtypes in TCGA and KBC datasets: a) *ESR1*/*ESR2* ratios; b) age at diagnosis; c) 10-year overall survival. **Figure S8.** Expression of *APOBEC3B* in normal and tumor tissue in relation to the polymorphic germline *APOBEC3B* deletion represented by rs12628403-C allele in HKBC. **Figure S9.** MCP-counter scores of eight immune cell subpopulations in adjacent normal breast tissue in the three luminal immune subgroups and non-luminal (HER2-enriched and basal-like) patients of HKBC.

**Additional file 2: Table S1.** The distribution of clinical characteristics and key breast cancer risk factors in the Hong Kong breast cancer study (HKBC). **Table S2.** 130 immune-related genes used for the classification of luminal tumors. **Table S3.** The three luminal immune subtypes in relation to the luminal A/B classification in the Hong Kong breast cancer study (HKBC). **Table S4.** *P* values for comparisons of tumor MCP-counter abundance scores by immune subtype in the Hong Kong breast cancer study (HKBC). **Table S5.** *P* values for comparisons of tumor CIBERSORT fraction scores by immune subtype in the Hong Kong breast cancer study (HKBC). **Table S6.** The distribution of clinical characteristics and key breast cancer risk factors by luminal immune subtypes in the Hong Kong breast cancer study (HKBC). **Table S7.** P values for comparisons of the MCP-counter abundance scores between paired tumor and normal tissue (*N* = 80) by immune subtype in the Hong Kong breast cancer study (HKBC).

## Data Availability

The datasets used and/or analyzed during the current study are available from the corresponding author on reasonable request.

## Abbreviations

BC: Breast cancer
HKBC: BC patients in Hong Kong
KBC: Korean breast cancer study
TCGA: The Cancer Genome Atlas
TILs: Infiltrating lymphocytes
TNBC: Triple-negative breast cancer
