# Supplementary material for: Immune gene expression profiling reveals heterogeneity in luminal breast tumors
Source: Breast Cancer Res. 2019 Dec 19;21:147. doi: 10.1186/s13058-019-1218-9 (PMC6924001; doi:10.1186/s13058-019-1218-9)
Supplement: Supplementary file 1 — Additional file 1: Figure S1. Schematic representation of the strategy for the quantification of TILs in H&E stained images. Figure S2. Expression levels of immune-related genes in the three luminal immune subtypes and non-luminal (HER2-enriched and basal-like) tumors in HKBC. Figure S3. MCP-counter scores and CIBERSORT relative fractions of a subset of immune cell subpopulations in the three luminal immune subtypes and non-luminal (HER2-enriched and basal-like, separately) tumors in HKBC. Figure S4. MCP-counter scores of eight immune cell subpopulations in the three luminal immune subtypes and non-luminal (HER2-enriched and basal-like) tumors with the adjustment of tumor purity in HKBC. Figure S5. Replication of luminal immune subtypes in TCGA and KBC datasets: a) MCP-counter scores for eight immune cell subpopulations; b) Relative fractions of immune cell subpopulations by CIBERSORT (cell populations with extremely low fractions were not shown); c) High-TIL tumors showed upregulation of genes in immune activation and regulation activities than tumors in the other two luminal immune subtypes; d) High-ISG tumors expressed higher levels of ISG genes than tumors in the other two luminal immune subtypes. Figure S6. Genomic features associated with luminal immune subtypes in HKBC (HER2-enriched and basal-like, separately): a) ESR1/ESR2 ratios; b) number of mutations. Figure S7. Replication of genomic features associated with luminal immune subtypes in TCGA and KBC datasets: a) ESR1/ESR2 ratios; b) age at diagnosis; c) 10-year overall survival. Figure S8. Expression of APOBEC3B in normal and tumor tissue in relation to the polymorphic germline APOBEC3B deletion represented by rs12628403-C allele in HKBC. Figure S9. MCP-counter scores of eight immune cell subpopulations in adjacent normal breast tissue in the three luminal immune subgroups and non-luminal (HER2-enriched and basal-like) patients of HKBC. [file 13058_2019_1218_MOESM1_ESM.docx]

**Supplementary Material (figures)**

**Figure S1** – Schematic representation of the strategy for the quantification of TILs in H&E stained images.

**Figure S2** - Expression levels of immune-related genes in the three luminal immune subtypes and non-luminal (HER2-enriched and basal-like) tumors in HKBC.

**Figure S3** - MCP-counter scores and CIBERSORT relative fractions of a subset of immune cell subpopulations in the three luminal immune subtypes and non-luminal (HER2-enriched and basal-like, separately) tumors in HKBC.

**Figure S4** - MCP-counter scores of eight immune cell subpopulations in the three luminal immune subtypes and non-luminal (HER2-enriched and basal-like) tumors with the adjustment of tumor purity in HKBC.

**Figure S5** - Replication of luminal immune subtypes in TCGA and KBC datasets: a) MCP-counter scores for eight immune cell subpopulations; b) Relative fractions of immune cell subpopulations by CIBERSORT (cell populations with extremely low fractions were not shown); c) High-TIL tumors showed upregulation of genes in immune activation and regulation activities than tumors in the other two luminal immune subtypes; d) High-ISG tumors expressed higher levels of ISG genes than tumors in the other two luminal immune subtypes.

**Figure S6** - Genomic features associated with luminal immune subtypes in HKBC (HER2-enriched and basal-like, separately): a) *ESR1*/*ESR2* ratios; b) number of mutations.

**Figure S7** - Replication of genomic features associated with luminal immune subtypes in TCGA and KBC datasets: a) *ESR1*/*ESR2* ratios; b) age at diagnosis; c) 10-year overall survival.

**Figure S8** - Expression of *APOBEC3B* in normal and tumor tissue in relation to the polymorphic germline *APOBEC3B* deletion represented by rs12628403-C allele in HKBC.

**Figure S9** - MCP-counter scores of eight immune cell subpopulations in adjacent normal breast tissue in the three luminal immune subgroups and non-luminal (HER2-enriched and basal-like) patients of HKBC.


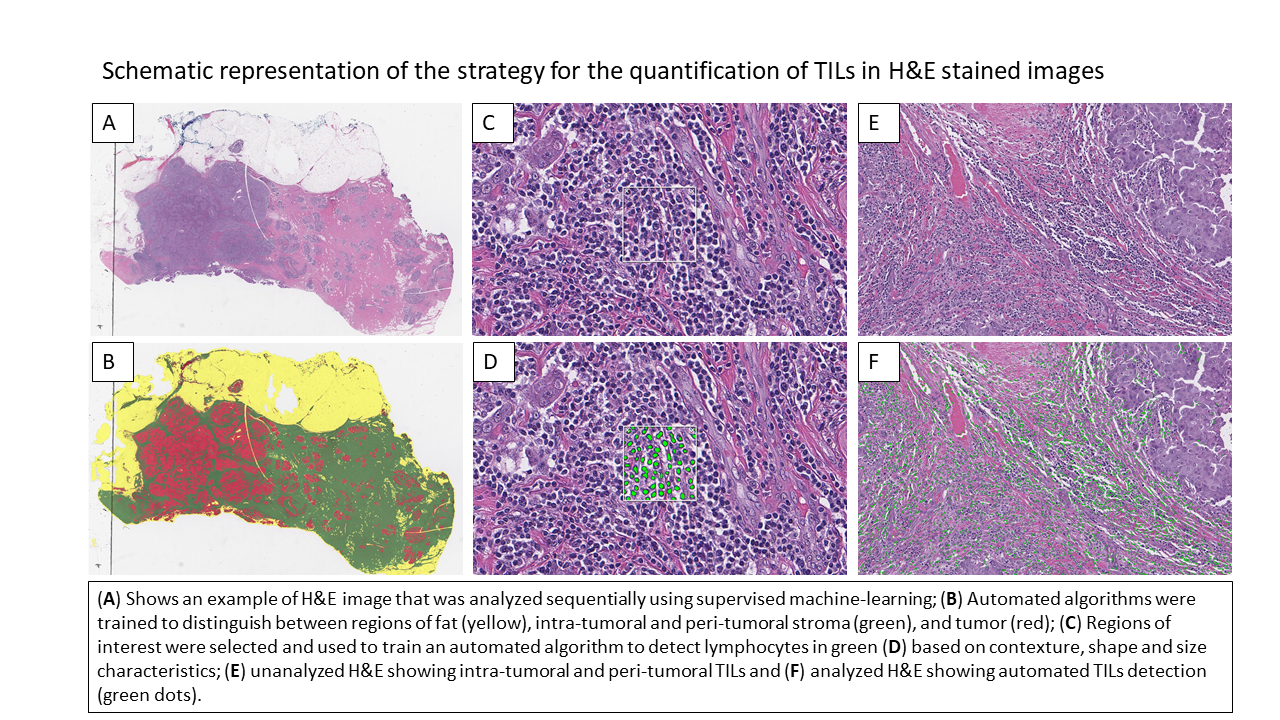


**Figure S1**


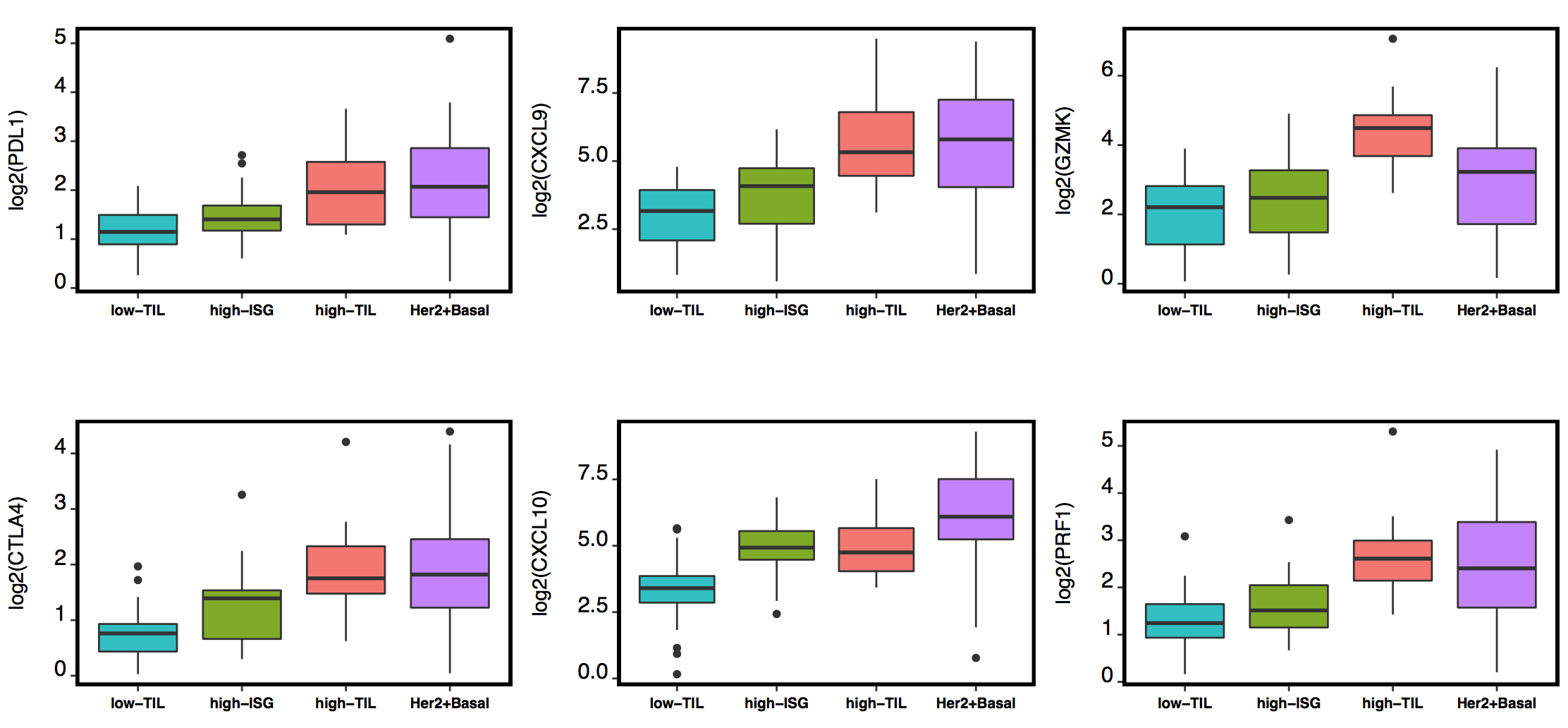


**Figure S2**


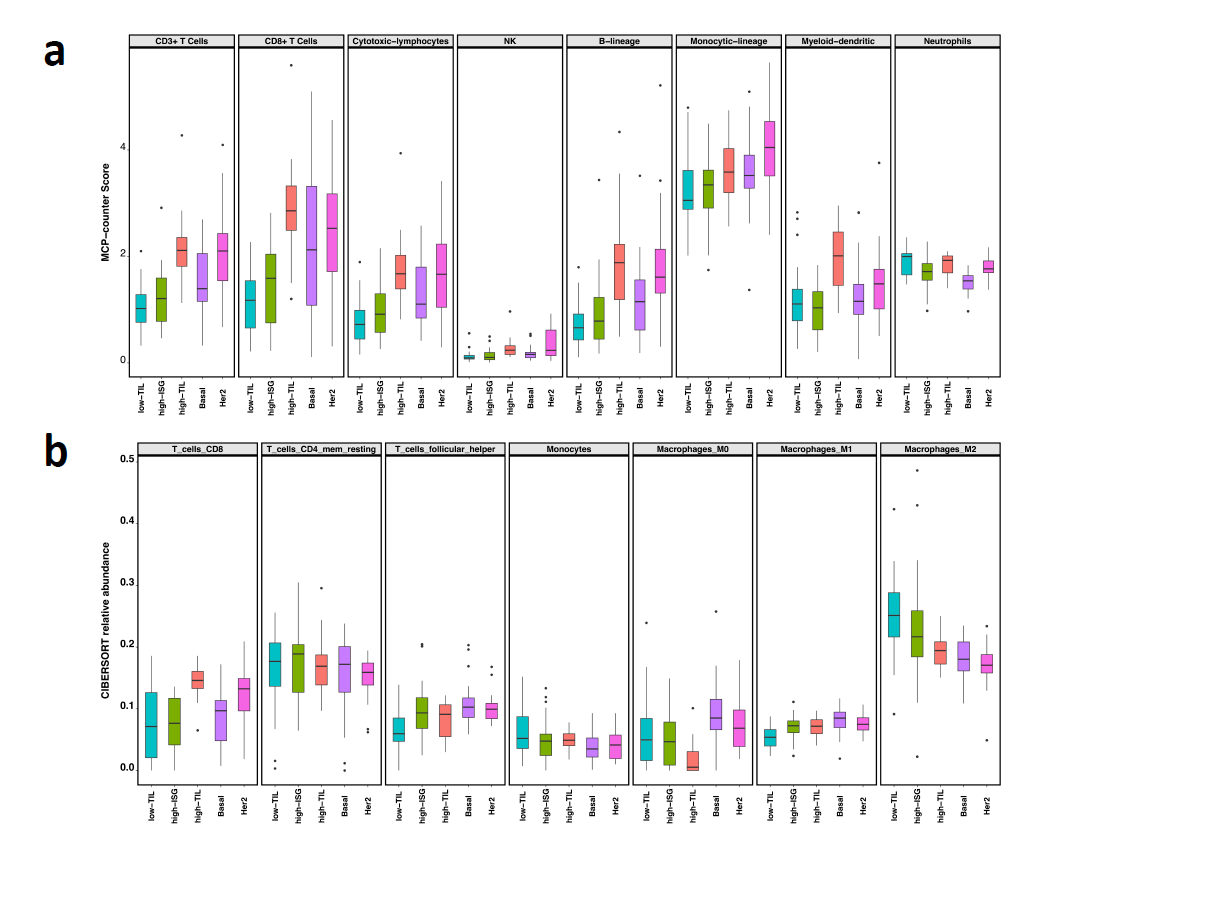


**Figure S3**


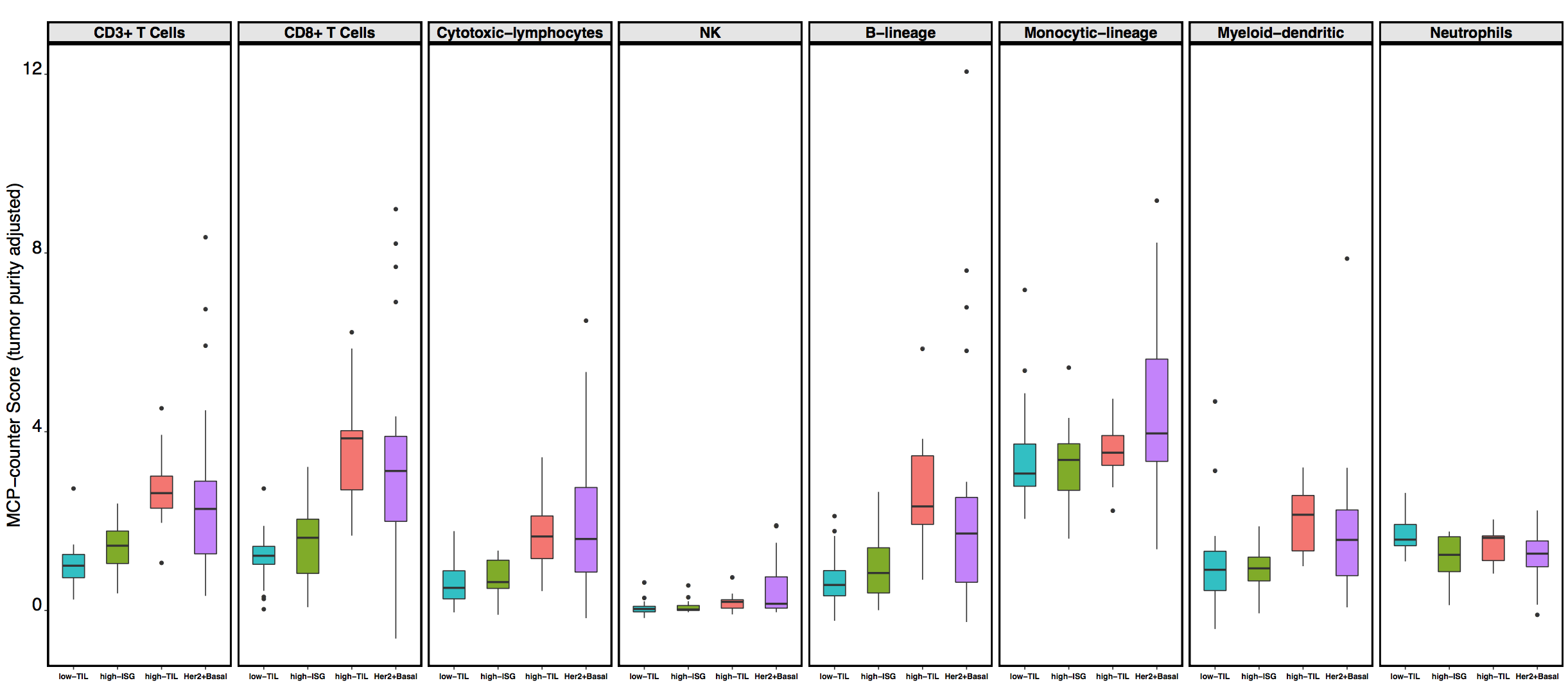


**Figure S4**

**TCGA Asian**


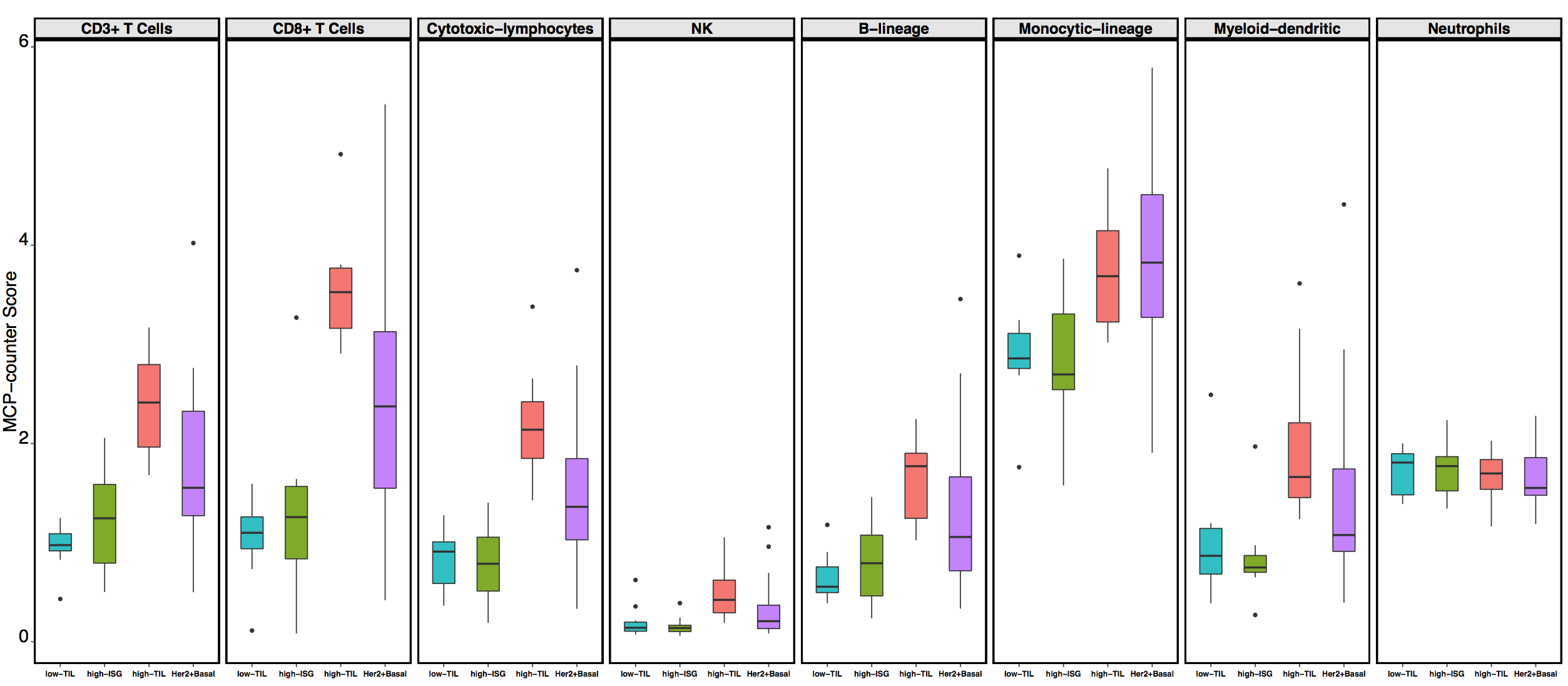


**TCGA White**


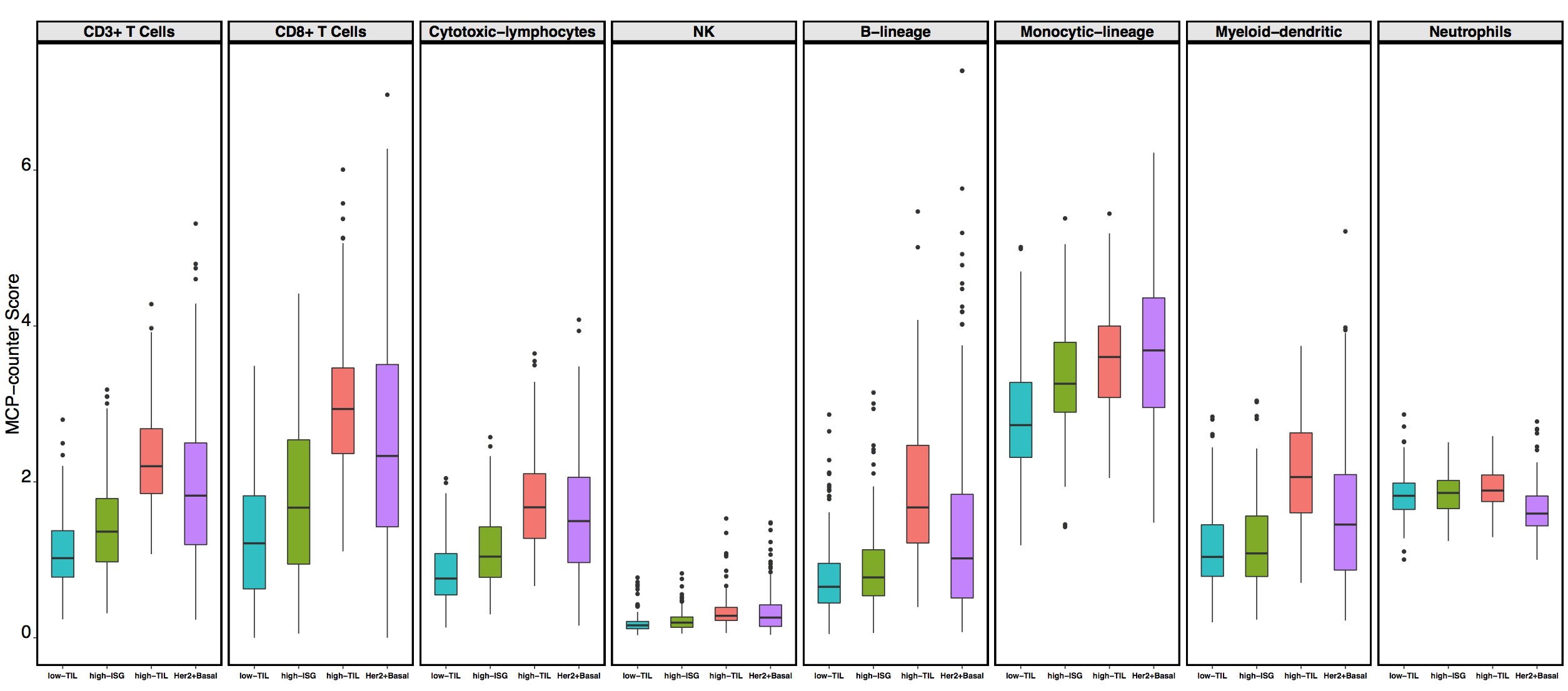


**Figure S5.a (*to be continued*)**

**TCGA Black**


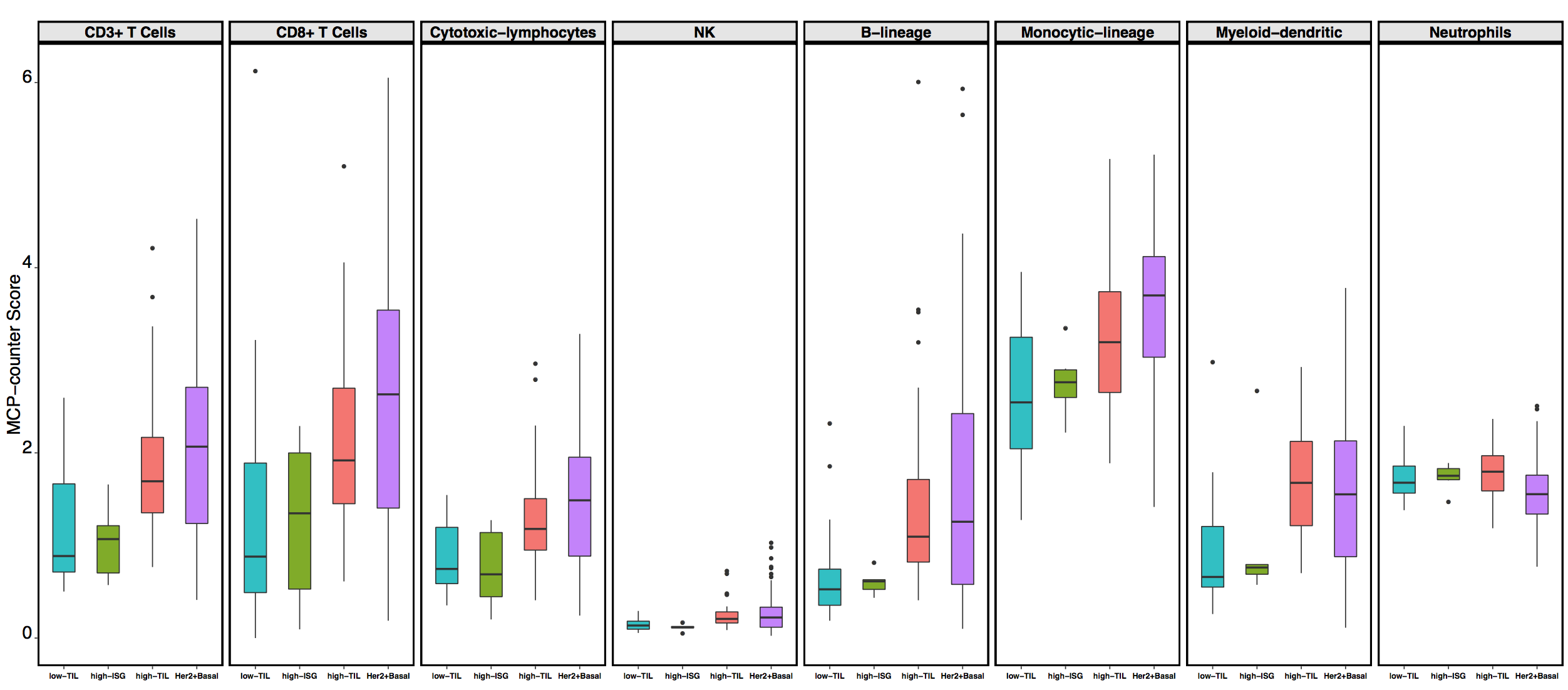


**KBC**


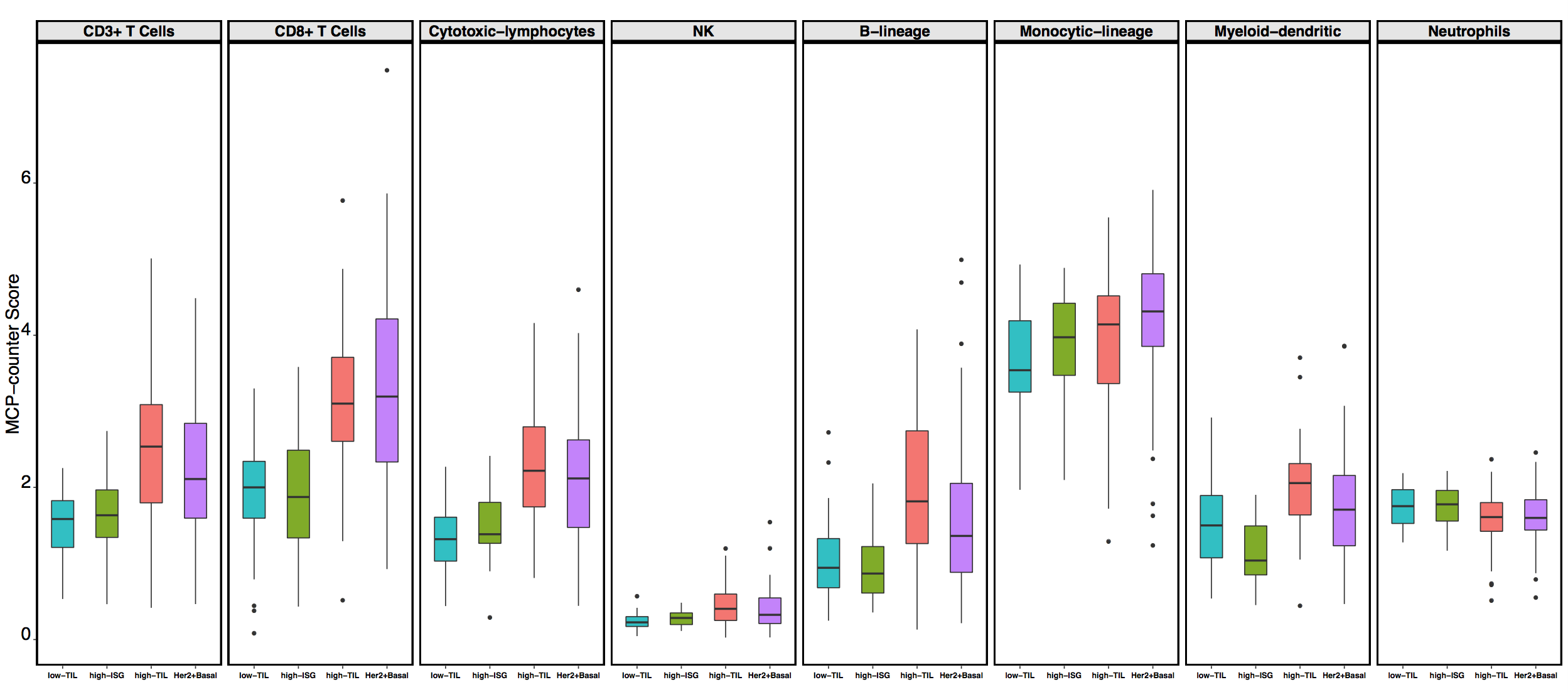


**Figure S5.a**

**TCGA Asian**


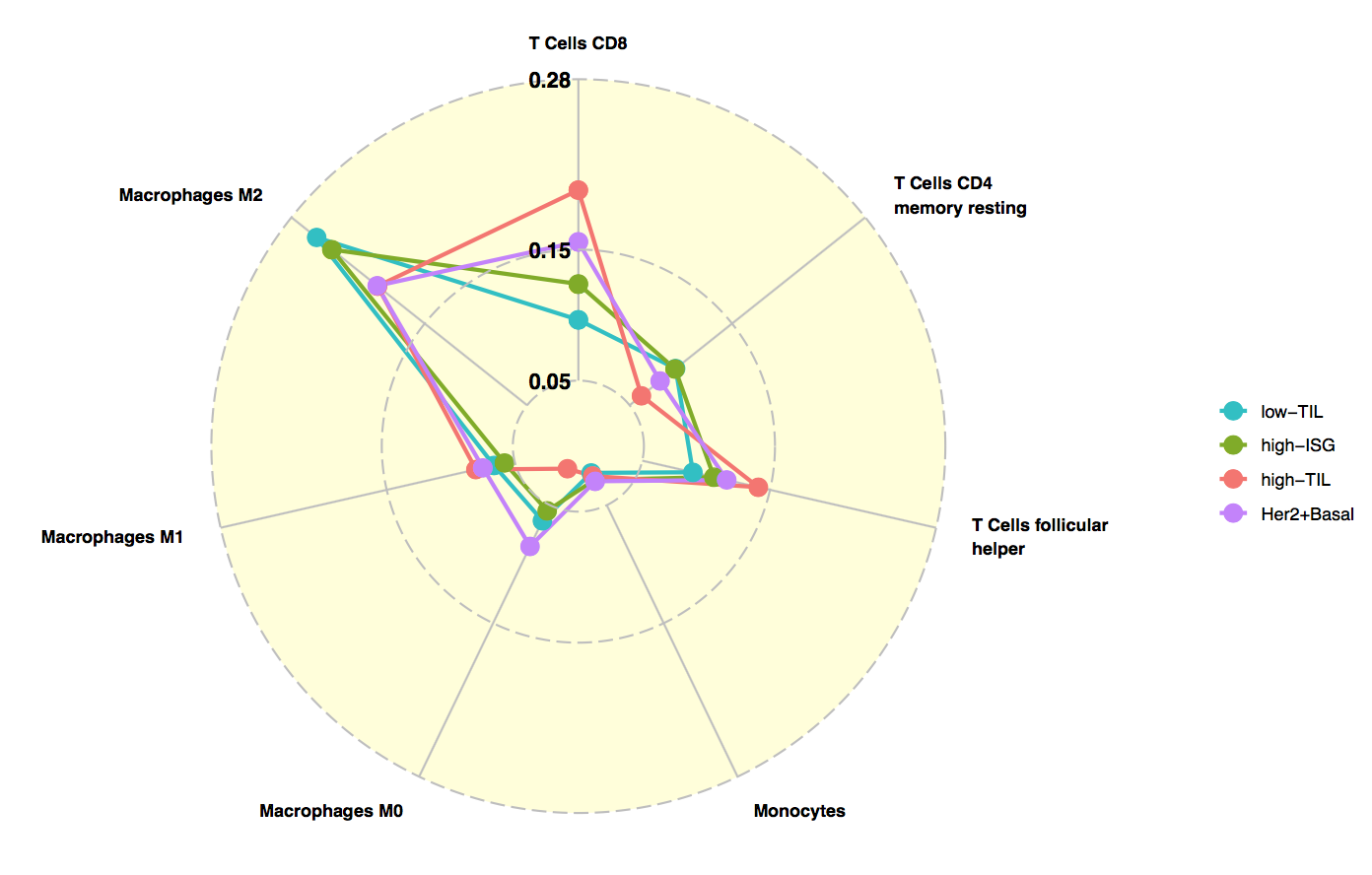


**TCGA White**


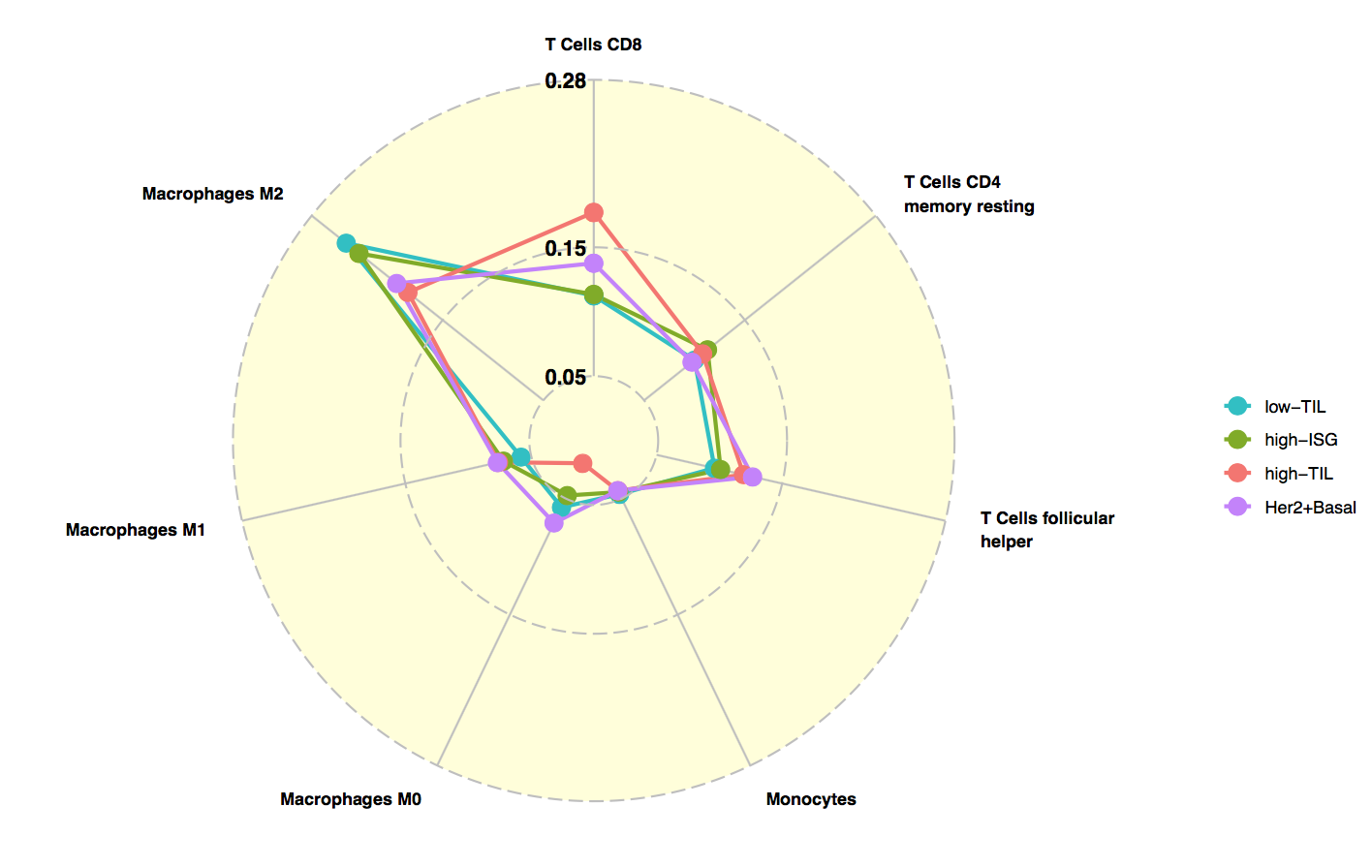


**Figure S5.b (*to be continued*)**

**TCGA Black**


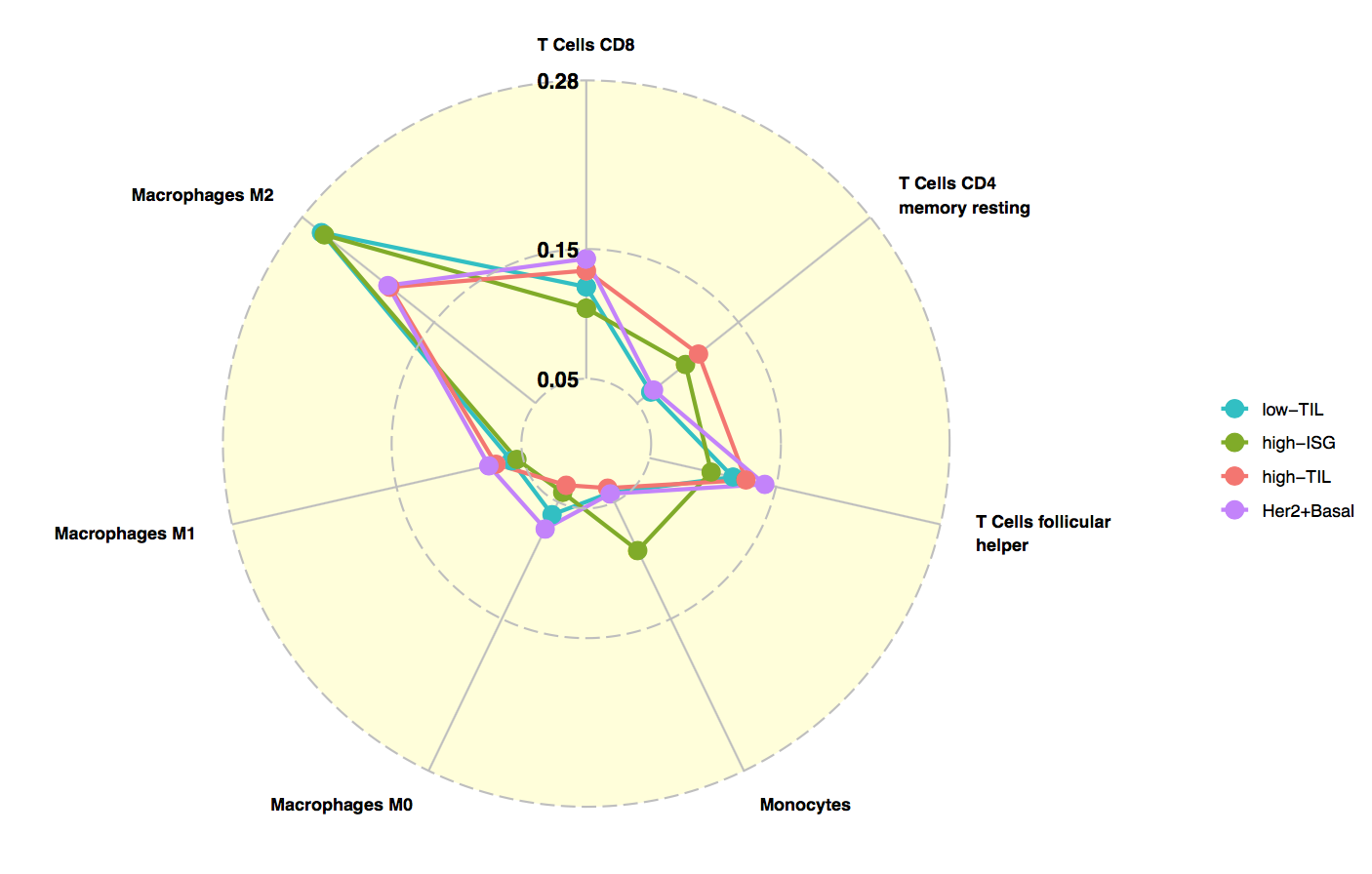


**KBC**


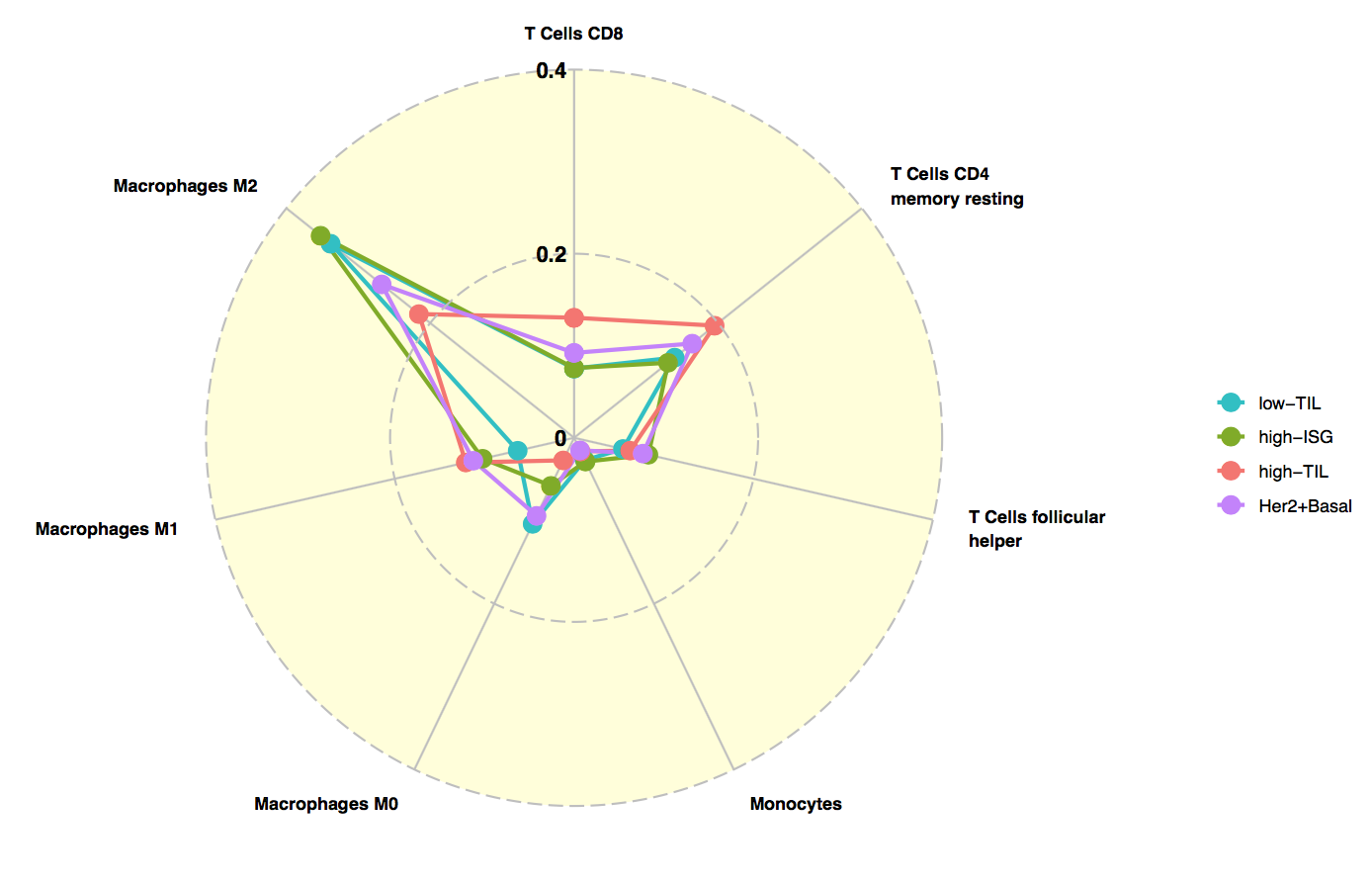


**Figure S5.b**

**TCGA**


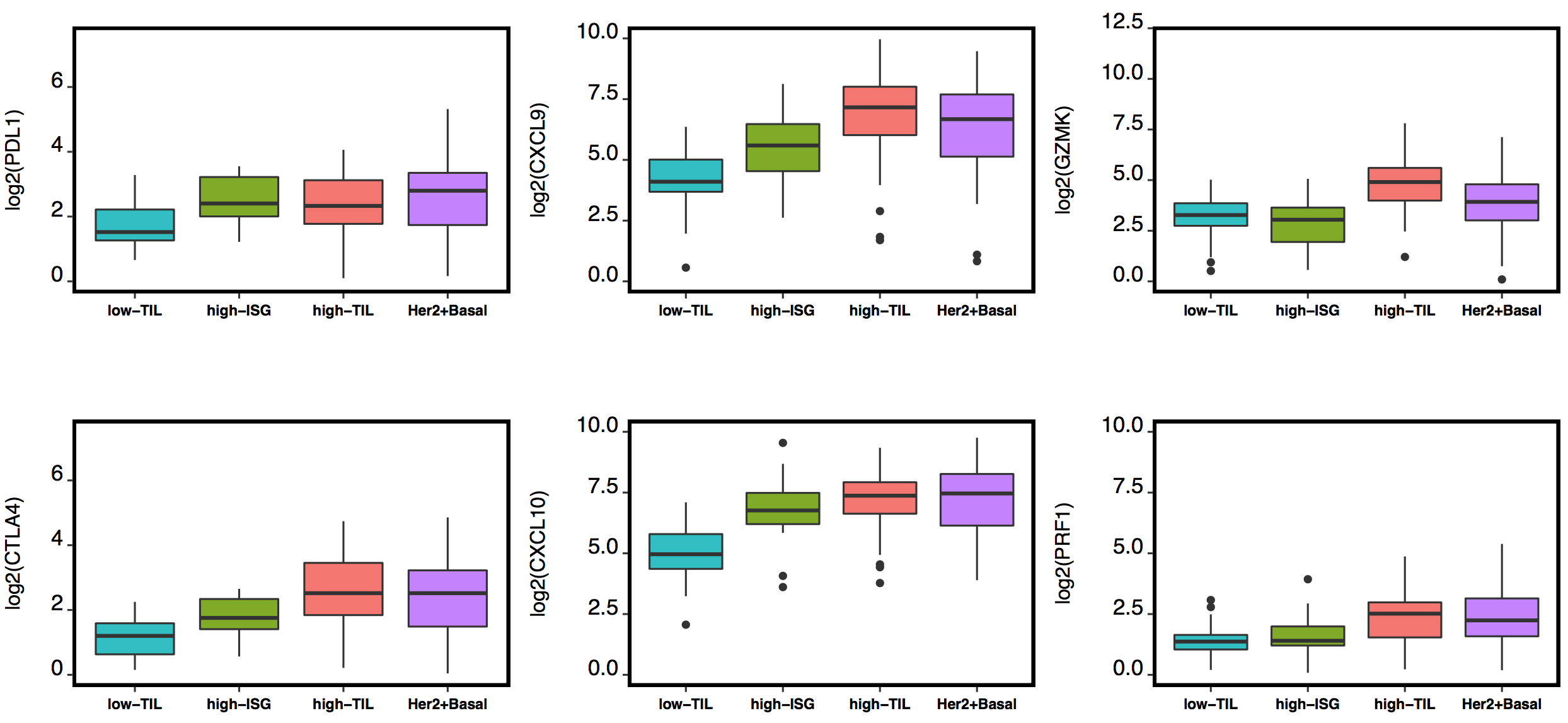


**KBC**


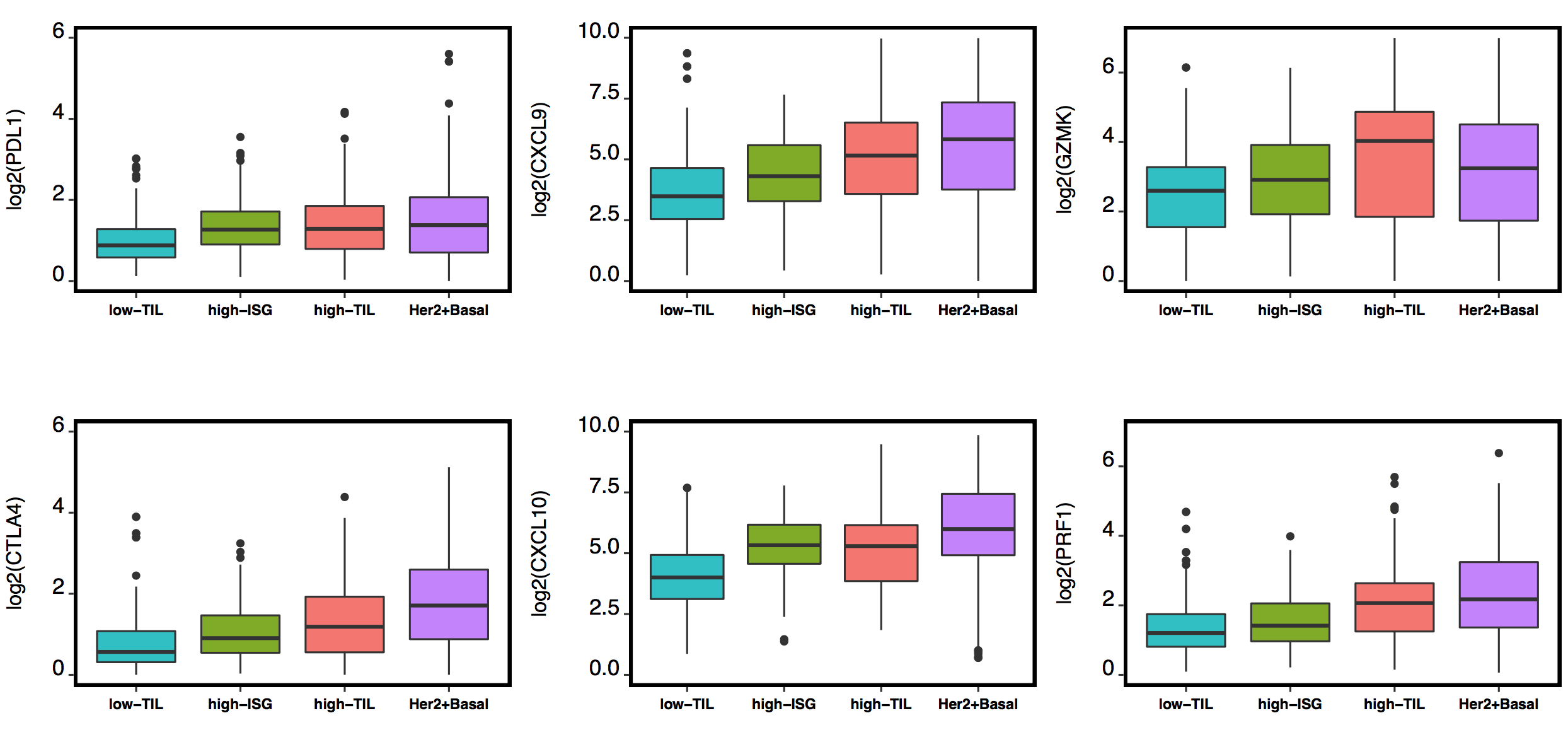


**Figure S5.c**


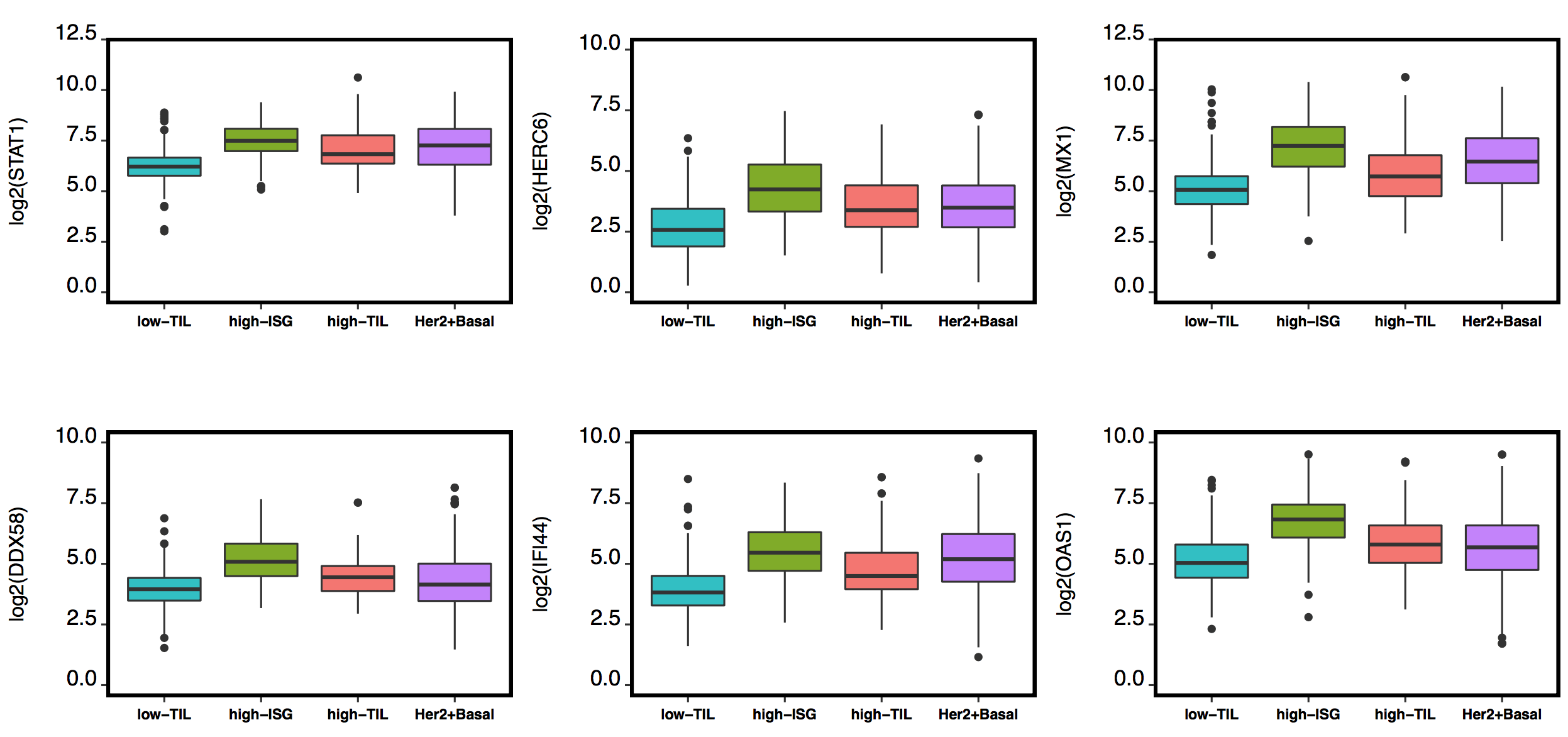


**TCGA**

**KBC**


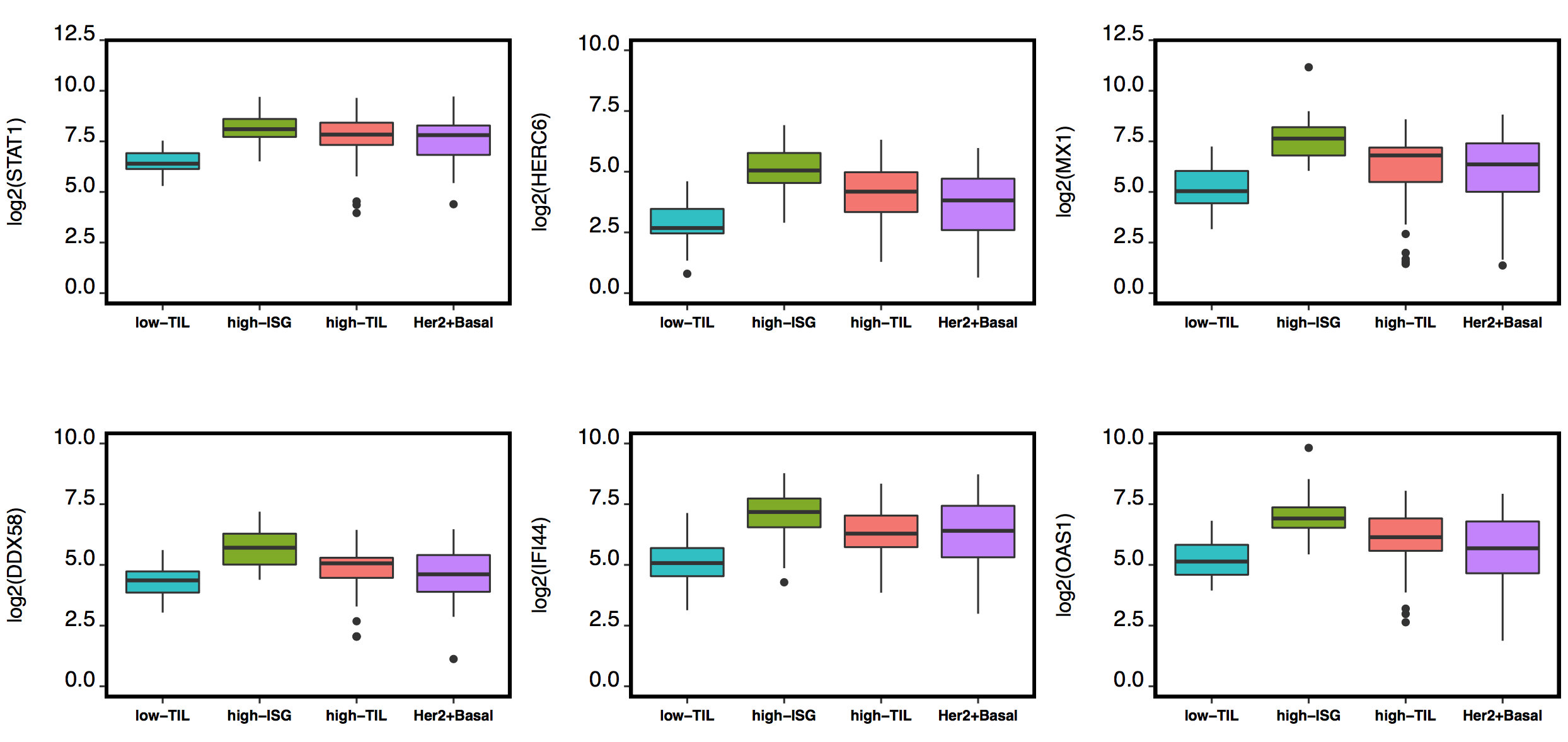


**Figure S5.d**


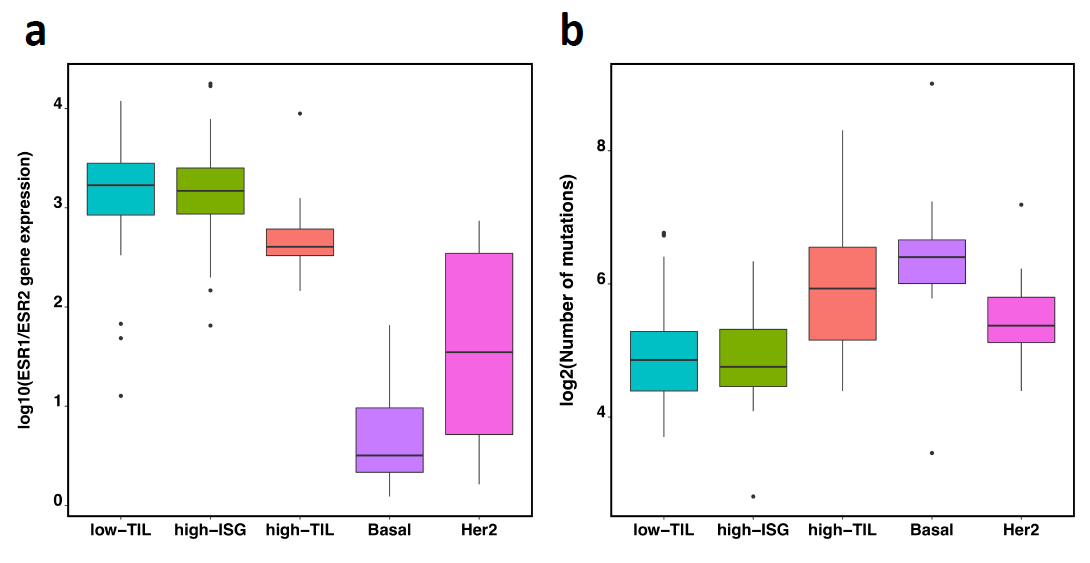


**Figure S6**


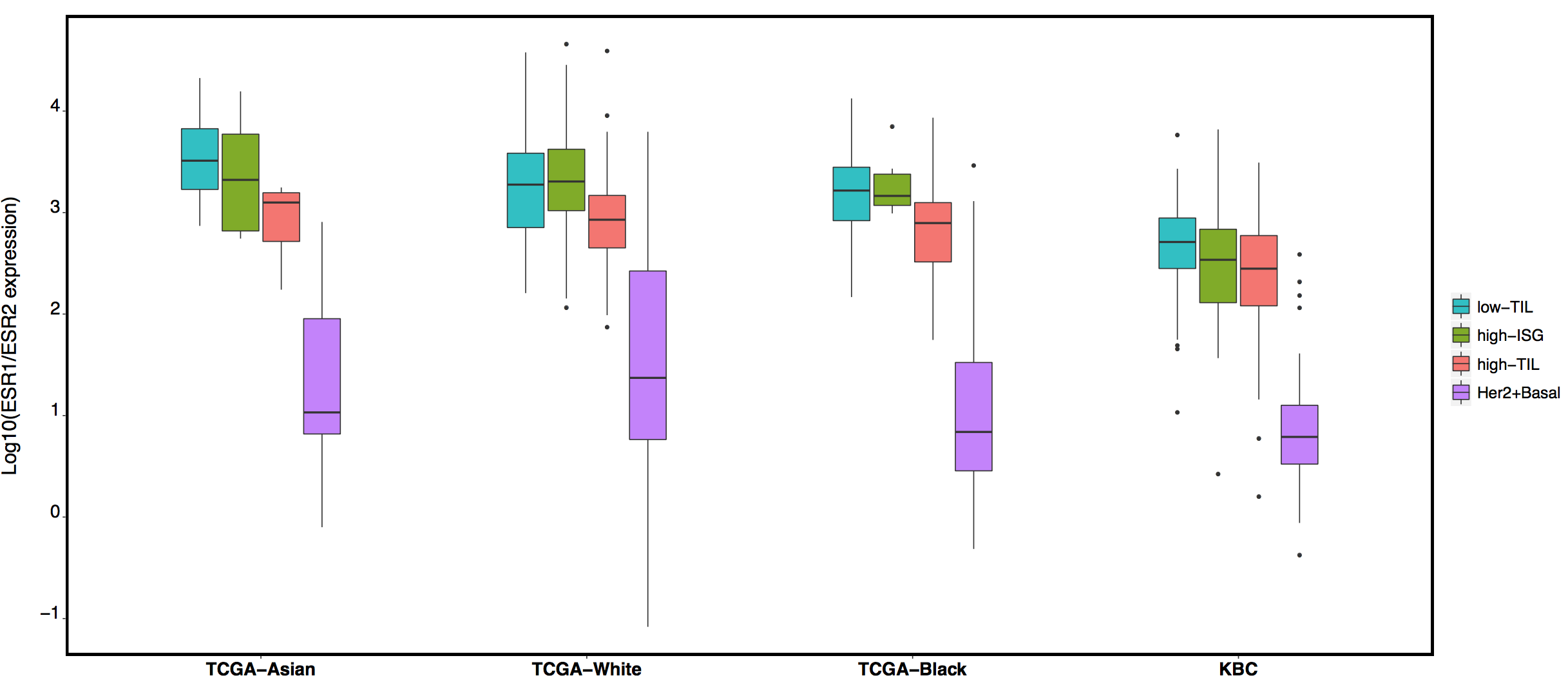


**Figure S7.a**


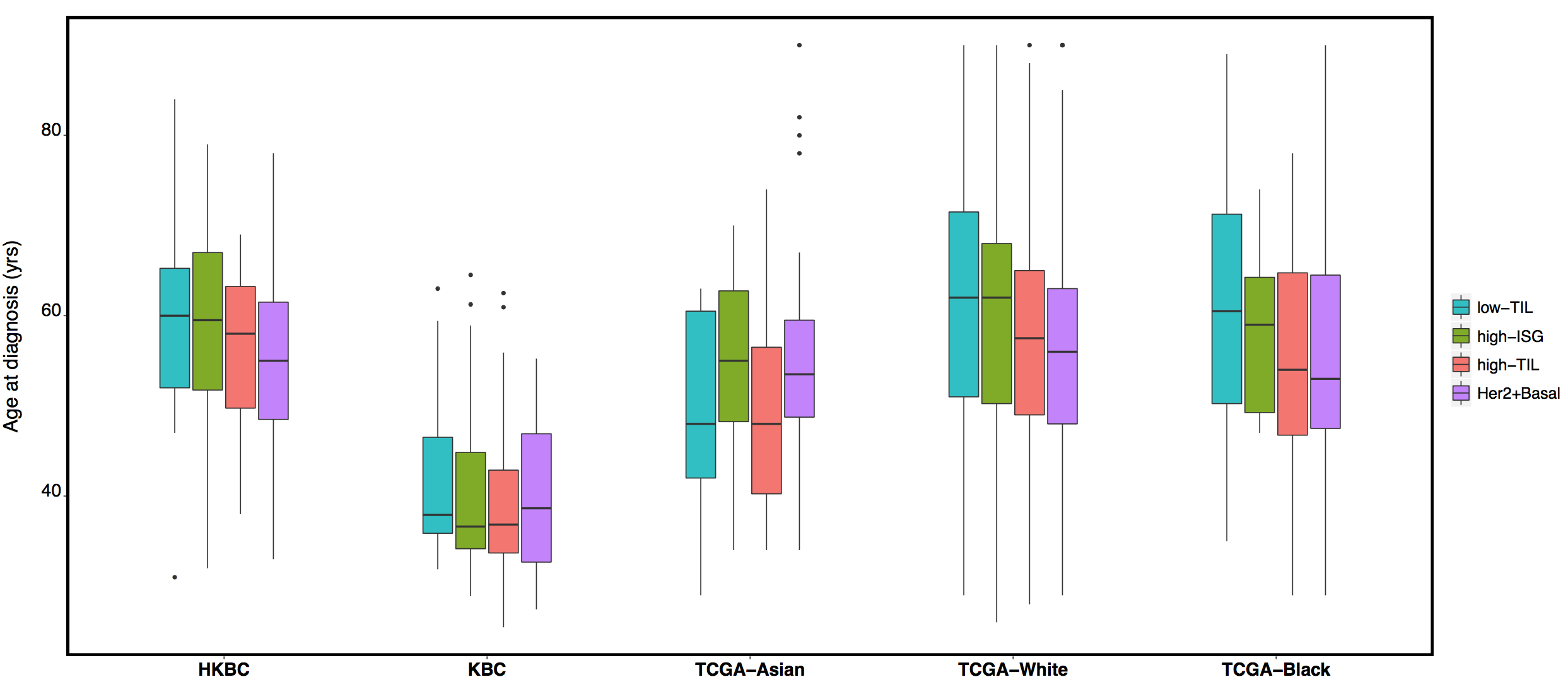


**Figure S7.b**

**TCGA BRCA 10-year survival**


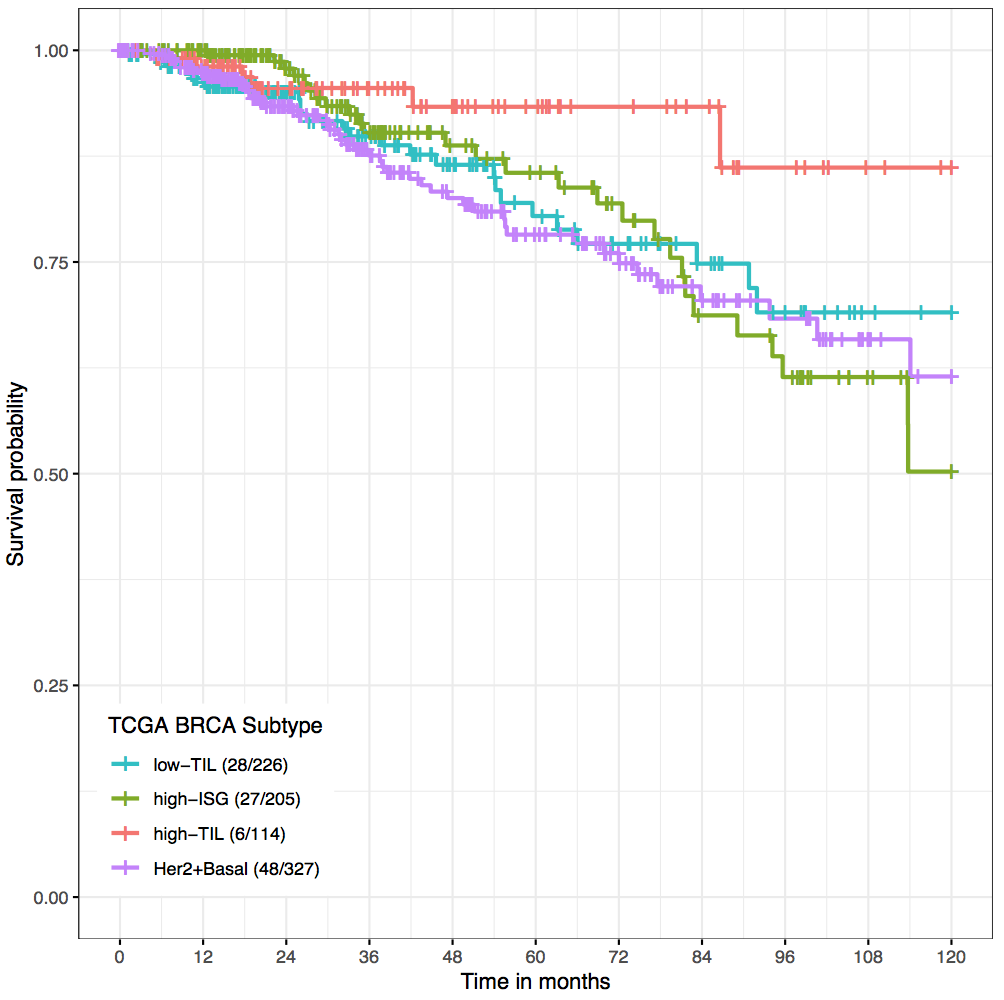


872 samples


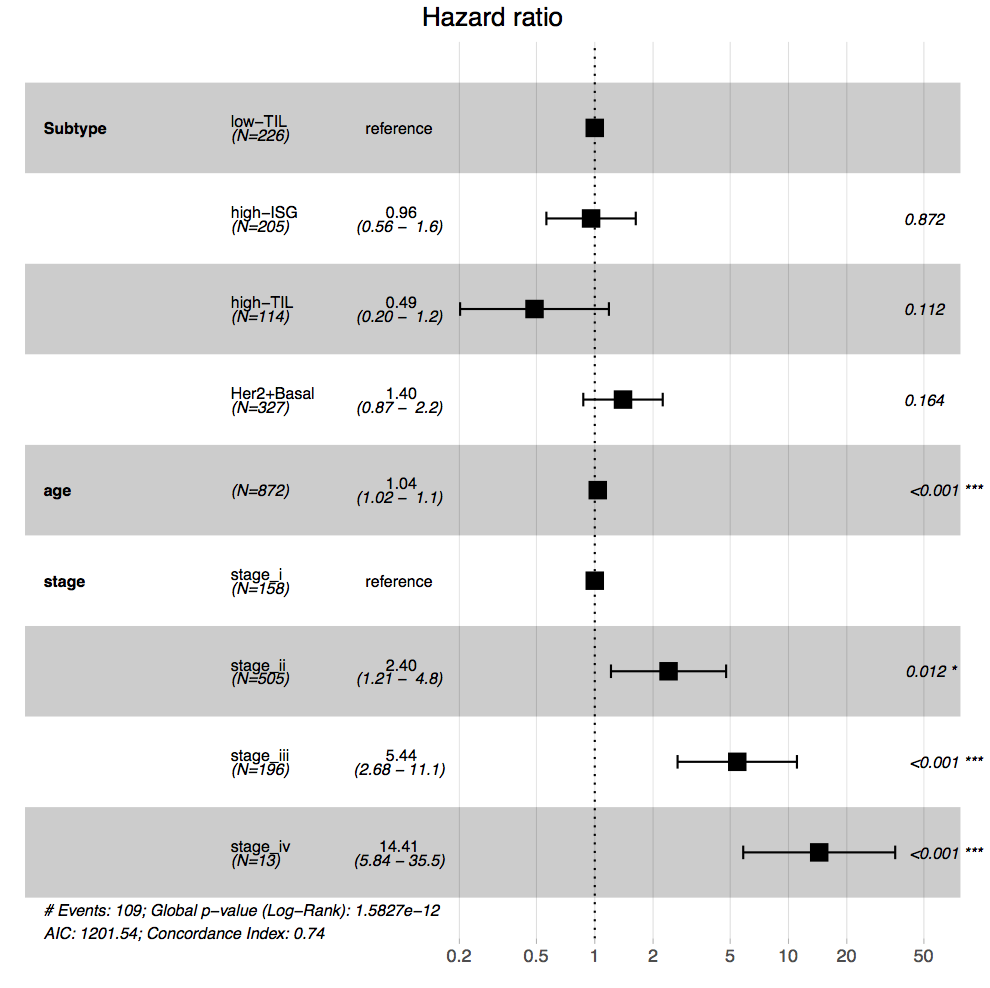


**Figure S7.c**


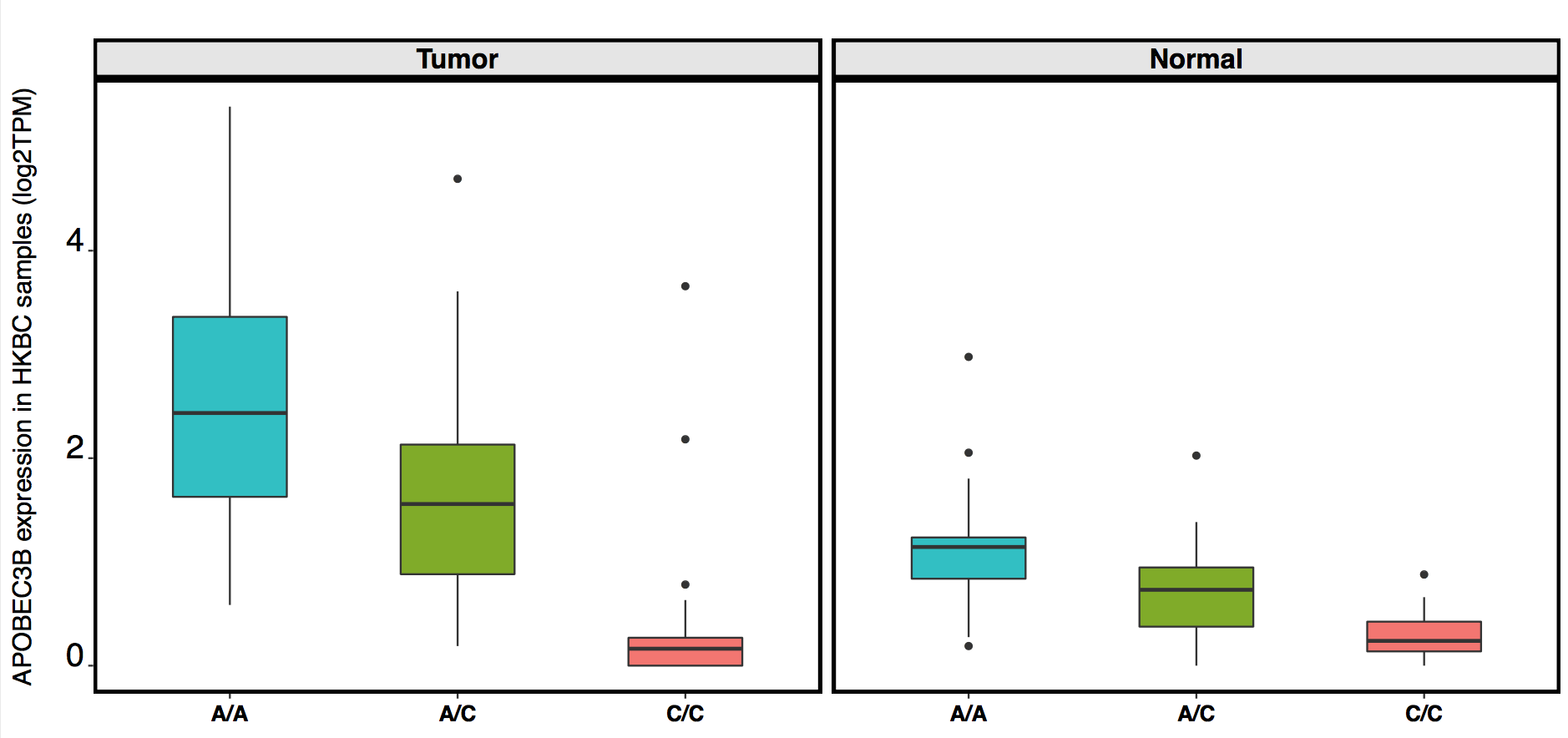


**Figure S8**


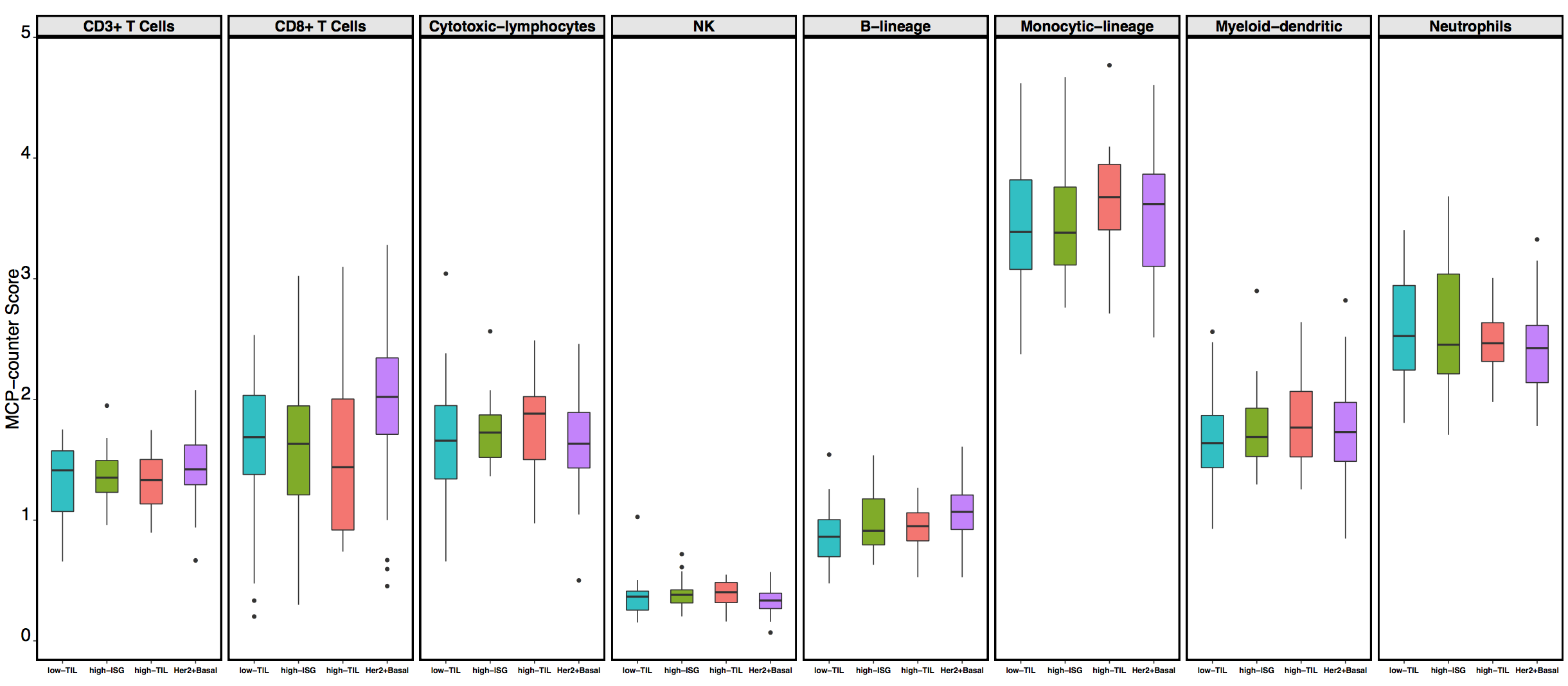


**Figure S9**
