# Supplementary material for: Immune gene expression profiling reveals heterogeneity in luminal breast tumors
Source: Breast Cancer Res. 2019 Dec 19;21:147. doi: 10.1186/s13058-019-1218-9 (PMC6924001; doi:10.1186/s13058-019-1218-9)
Supplement: Supplementary file 2 — Additional file 2: Table S1. The distribution of clinical characteristics and key breast cancer risk factors in the Hong Kong breast cancer study (HKBC). Table S2. 130 immune-related genes used for the classification of luminal tumors. Table S3. The three luminal immune subtypes in relation to the luminal A/B classification in the Hong Kong breast cancer study (HKBC). Table S4. P values for comparisons of tumor MCP-counter abundance scores by immune subtype in the Hong Kong breast cancer study (HKBC). Table S5. P values for comparisons of tumor CIBERSORT fraction scores by immune subtype in the Hong Kong breast cancer study (HKBC). Table S6. The distribution of clinical characteristics and key breast cancer risk factors by luminal immune subtypes in the Hong Kong breast cancer study (HKBC). Table S7. P values for comparisons of the MCP-counter abundance scores between paired tumor and normal tissue (N = 80) by immune subtype in the Hong Kong breast cancer study (HKBC). [file 13058_2019_1218_MOESM2_ESM.docx]

**Supplementary Material (tables)**

**Table S1** - The distribution of clinical characteristics and key breast cancer risk factors in the Hong Kong breast cancer study (HKBC)

**Table S2** - 130 immune-related genes used for the classification of luminal tumors

**Table S3** - The three luminal immune subtypes in relation to the luminal A/B classification in the Hong Kong breast cancer study (HKBC)

**Table S4** - P values for comparisons of tumor MCP-counter abundance scores by immune subtype in the Hong Kong breast cancer study (HKBC)

**Table S5** - P values for comparisons of tumor CIBERSORT fraction scores by immune subtype in the Hong Kong breast cancer study (HKBC)

**Table S6** - The distribution of clinical characteristics and key breast cancer risk factors by luminal immune subtypes in the Hong Kong breast cancer study (HKBC)

**Table S7** - P values for comparisons of the MCP-counter abundance scores between paired tumor and normal tissue (N=80) by immune subtype in the Hong Kong breast cancer study (HKBC)

low-TIL(Lum1), high-ISG(Lum2), high-TIL(Lum3)

* p-values using one-way analysis of variance (ANOVA) for continuous variables

or chi-square tests for categorical variables

* p-values using one-way analysis of variance (ANOVA) for continuous variables

or chi-square tests for categorical variables

low-TIL(Lum1), high-ISG(Lum2), high-TIL(Lum3)

^a^ p-values were obtained from age and BMI adjusted logistic regression models

**anything in boldface represents a significant result**

^a^ p-values were obtained from age and BMI adjusted logistic regression models

**anything in boldface represents a significant result**

low-TIL(Lum1), high-ISG(Lum2), high-TIL(Lum3)

* p-values were obtained from one-way analysis of variance (ANOVA) for continuous variables or chi-square tests for categorical variables

^a^ p-values were obtained from comparing tumor and normal samples using matched t-test

**anything in boldface represents a significant result**
